# Implementation of a Hamming distance–like genomic quantum classifier using inner products on ibmqx2 and ibmq_16_melbourne

**DOI:** 10.1007/s42484-020-00017-7

**Published:** 2020-07-17

**Authors:** Kunal Kathuria, Aakrosh Ratan, Michael McConnell, Stefan Bekiranov

**Affiliations:** 1 Department of Biochemistry and Molecular Genetics, University of Virginia, Charlottesville, VA USA; 2 Center for Public Health Genomics, University of Virginia, Charlottesville, VA USA

**Keywords:** Quantum classifier, Genomic classifier, Quantum machine learning, Quantum algorithms

## Abstract

Motivated by the problem of classifying individuals with a disease versus controls using a functional genomic attribute as input, we present relatively efficient general purpose inner product–based kernel classifiers to classify the test as a normal or disease sample. We encode each training sample as a string of 1 s (presence) and 0 s (absence) representing the attribute’s existence across ordered physical blocks of the subdivided genome. Having binary-valued features allows for highly efficient data encoding in the computational basis for classifiers relying on binary operations. Given that a natural distance between binary strings is Hamming distance, which shares properties with bit-string inner products, our two classifiers apply different inner product measures for classification. The active inner product (AIP) is a direct dot product–based classifier whereas the symmetric inner product (SIP) classifies upon scoring correspondingly matching genomic attributes. SIP is a strongly Hamming distance–based classifier generally applicable to binary attribute-matching problems whereas AIP has general applications as a simple dot product–based classifier. The classifiers implement an inner product between *N* = 2^*n*^ dimension test and train vectors using *n* Fredkin gates while the training sets are respectively entangled with the class-label qubit, without use of an ancilla. Moreover, each training class can be composed of an arbitrary number *m* of samples that can be classically summed into one input string to effectively execute all test–train inner products simultaneously. Thus, our circuits require the same number of qubits for any number of training samples and are $O(\log {N})$ in gate complexity after the states are prepared. Our classifiers were implemented on ibmqx2 (IBM-Q-team 2019b) and ibmq_16_melbourne (IBM-Q-team 2019a). The latter allowed encoding of 64 training features across the genome.

**Electronic supplementary material:**

The online version of this article (10.1007/s42484-020-00017-7) contains supplementary material, which is available to authorized users.

## Introduction

Quantum computing algorithms have been developed that show great promise of making potentially significant improvements upon existing classical equivalents, particularly in the area of machine learning. Exponential speedups have been predicted for implementing least squares fitting (Wiebe et al. 2012), quantum Boltzmann machines (Amin et al. 2018; Kieferová and Wiebe 2017), quantum principal components analysis (Lloyd et al. 2014), and quantum support vector machines (Spagnolo et al. 2013) on a quantum computer, over their classical counterparts (Biamonte et al. 2017). Quadratic speedups have been theoretically demonstrated for Bayesian inference (Low et al. 2014; Wiebe and Granade 2015), online perceptron (Kapoor et al. 2016), classical Boltzmann machines (Wiebe et al. 2014b), and quantum reinforcement learning (Dunjko et al. 2016; Biamonte et al. 2017). However, these speedups presume a low-error rate, universal, quantum computer with hundreds to thousands of qubits. In addition, the speedup of a subset of these algorithms (e.g., quantum support vector machines) requires quantum RAM (qRAM) (Giovannetti et al. 2008), which would enable a quantum coherent mapping of a classical vector into a quantum state (Rebentrost et al. 2014), but this does not currently exist. Recent progress has been made in exploiting a relatively natural connection between kernel-based classification and quantum computing (Schuld et al. 2017; Havlícek et al. 2019; Schuld and Killoran 2019). One set of approaches maps a large feature space to a quantum state space in order to compute a kernel function on a quantum computer which is then used to optimally classify input data using, for example, a support vector machine (SVM) (Havlícek et al. 2019; Schuld and Killoran 2019). In another set of approaches, variational quantum circuits are used to directly classify the data in the large quantum feature space on a quantum computer, similar to the approach employed by an SVM (Schuld and Killoran 2019; Havlícek et al. 2019). An even more direct and simple implementation of kernel-based classification on quantum computers exploit quantum interference of the training and test vectors which when properly prepared as quantum states execute distance-based classification upon measurement (Schuld et al. 2017; Schuld et al. 2014a). An advantage of these kernel-based approaches is that they can and have been implemented on existing quantum computers. However, in the case of the quantum interference circuit (Schuld et al. 2017), the distance measure was Euclidean-based and only two training vectors could be used to execute the classifier on a 5-qubit processor.

Motivated by the problem of classifying individuals with a disease (e.g., Alzheimer’s disease) given single cell neuronal genomic copy number variation (CNV) data (van den Bos et al. 2016; McConnell et al. 2013; McConnell et al. 2017; Chronister et al. 2019), we developed a set of quantum classifier circuits which exploit a biologically relevant encoding of the training and test vectors that allow us to take full advantage of the computational basis of the quantum computer. Specifically, the training and test vectors encode the presence and absence of a CNV in a given genomic window with a 1 and 0, respectively, in non-overlapping windows across the human genome (i.e., they can be represented as binary strings). These genomic windows, which are nothing but ordered physical subdivisions of the genome, act as our feature dimensions (more precisely, the CNVs in respective genomic regions constitute the different features). The classification is based directly on the highest inner product between the test and training set of each class. This is possible because the class label qubit (which is eventually measured) is directly entangled with the training vectors, rendering the usual ancilla qubit unnecessary. Furthermore, we take advantage of many of the shared properties between a natural distance measure between binary strings, the Hamming distance, and the inner product between vectors composed of many binary strings to arrive at the simplest implementation of a near-term quantum disease-control classifier using genomic input data. The classifier is broad in its scope and can be applied to any situation amenable to using inner products in a feature space composed of divisions of physical space, time, etc., or of any correspondingly comparable attributes as discussed in the following sections. Notably, we are aware of two works which implement Hamming distance–based classification schemes (Ruan et al. 2017; Schuld et al. 2014b). In the former, k-nearest-neighbors (kNN) classification is implemented based on the metric of Hamming distance, which is essentially calculated by storing the difference between binary vector components of the test and training vectors in a register and adding up the negative difference over the vector dimensions. Only the nearest neighbors are used in classification, and the training vectors farther than a threshold distance from the test are neglected by the algorithm. Binary feature data is encoded in bit-vector form, thus using *n* qubits to encode *n* features. The Hamming distance between the test and each vector of the training set is calculated and iteratively summed over the *n* dimensions by adapting a circuit presented in Kaye (2004) to enable correct classification. An actual circuit is not implemented; however, the complexity was found to be *O*(*n*^3^). In the latter work, another Hamming distance–based kNN-like classifier using the same encoding scheme as Ruan et al. (2017) is developed to address a pattern recognition problem based on a proposal of Trugenberger (2001). In this work, the Hamming distance between the test and each training vector is eventually encoded as an amplitude into a register containing a superposition of the training vectors from each training set. The probability of measuring the class label qubit (entangled set-wise to the superposition above) in a particular state attempts to encode a monotonic function (a sum of square cosines over the training set) of the average Hamming distance of the test vector to the training set of the measured class. Each term of the said sum is certainly monotonic in its Hamming distance but it is not shown how the sum itself is monotonic in the average Hamming distance to the training vectors. The algorithm weighs lower distances between the test and potential neighbors more than higher ones, as with typical distance-based kNN classification (Dudani 1976). The class index measured with the highest probability is chosen to classify the test vector. An actual circuit is not drawn. Another somewhat related work is Wiebe et al. (2014a), which is a nearest-neighbor classifier that also employs the inner product in one approach to build half of its classification metric, the Euclidean vector distance. It does not implement the metric at the circuit level.

Our work executes an advance over the first two approaches above, in various ways. It presents two minimalistic, relatively efficient Hamming distance–based (SIP) and dot product–based (AIP) classifiers containing only two execution steps: data encoding and state overlap via swap test. Taking advantage of binarized encoding schemes (Schuld et al. 2015; Tacchino et al. 2019), single sample training data is binarized in genomic subdivisions and these binary training features are encoded as quantum state coefficients in the computational basis (similar to (Schuld et al. 2017) and detailed below), allowing us to encode and operate on 2^*n*^ *binary-valued* features with just *n* qubits. The class qubit is straightaway entangled with the training set with a minimal number of gates (2 in 14-qubit example problem 1 in “Results”) during data encoding and is measured at the end to directly reveal the correct class for the test vector. No ancilla qubit is used, and the inner product via swap test is coherently implemented using *n* Fredkin gates while the above qubits are entangled leading to *O*(*n*) complexity after state preparation. Moreover, the formulation of SIP is that the presence and absence of a CNV in a genomic region is encoded with a state coefficient of + 1 and − 1, respectively, in the computational basis, immediately yielding the total sum of bit matches minus mismatches after state overlap (which we show is equivalent to Hamming distance–based classification). As ours is not a kNN-like classifier, there is no distance-weighting bias in the algorithm and it is the simplest and most efficient implementation of directly using the total Hamming distance to the test as a classification measure that we are aware of. Furthermore, in our approach, we allow for the feature vector sum of the training set to be directly encoded in initial state preparation, thus allowing for the simultaneous evaluation of the similarity metric between the test and all the training vectors in one state overlap operation. Thus, in the process, we implement a linearized version of Hamming distance over multiple training inputs and use it for classification. Most importantly, we present an actual circuit implementation of our classifiers on both 5-qubit and 14-qubit hardware. The actual gate depth is *O*(*n*^3^) for *n* features (Ruan et al. 2017) or unclear (Schuld et al. 2014b) due to lack of an actual circuit implementation, but would be clearly greater when compared with our implementation of 5-qubit example problem 1 on ibmqx2 using 37 gates to arrive at the pre-final measurement state (including all swap operations necessary for the architecture and the 18 gates necessary for the standard swap test evaluation of state overlap (Online Resource 1)). Our classifiers have utility well beyond the genomic domain, as AIP can be used in any situation where a dot product between test and train scores similarity and SIP can be used in any situation which requires scoring the total bit-wise similarity between binary-valued feature vectors.

In summary, our circuits are employed to execute binary (disease/normal) classification of genomic samples on 5-qubit (ibmqx2) and 14-qubit (ibmq_16_melbourne) architectures using two different inner product metrics (Abraham et al. 2019). Genomics is an exciting rapidly advancing field that is much in need of effective machine learning solutions to keep up with fast-growing technological advancements in sequencing, the large amount of data generated, and the vast array of different relevant data types. Development of these and other near-term quantum algorithms will enable quantum computation to solve demanding, data-driven problems in genomics as we approach the development of powerful, low-error rate, universal quantum computers.

## Results and discussion

If one is interested in quantifying how well two arbitrary binary strings (of equal length *n*) match with each other, the most natural way would be to calculate their Hamming distance. The Hamming distance is simply the sum of the positional mismatches of the two bit strings. Thus, the Hamming distance of identical strings is 0 and that of two strings that are binary complements is *n*.

The Hamming distance has broad utility in solving classification problems that can use binary or binarized inputs representing a more complex event. Our classifier is equivalent to a Hamming distance–based classifier provided some caveats to the input data (see “Methods” for proof). As a simple warm-up example without any technical implementation details, we examine how our inner product–based classifier can be applied to a schedule-matching problem. There are certain time blocks in the day where individuals are either available or not available, and the goal is to determine which individual (or set of individuals) a given “test” individual’s schedule matches best (so they can find the most common time among all individual pairs to work together on a new issue, for example). Each time block is represented uniquely by a “block state” and a coefficient/prefactor attached to each such state represents a person’s availability during that time of day. For example, if we had 8 time blocks, we would represent them with 3 qubits, where the block state |000〉 would represent the first of these 8 blocks and the block state |111〉 would represent the last block. Thus, the time blocks constitute the feature space of the problem. In the simplest case, the state coefficients would be binary themselves (1 indicating the person’s availability and 0 indicating non-availability).

**Fig. 1:**
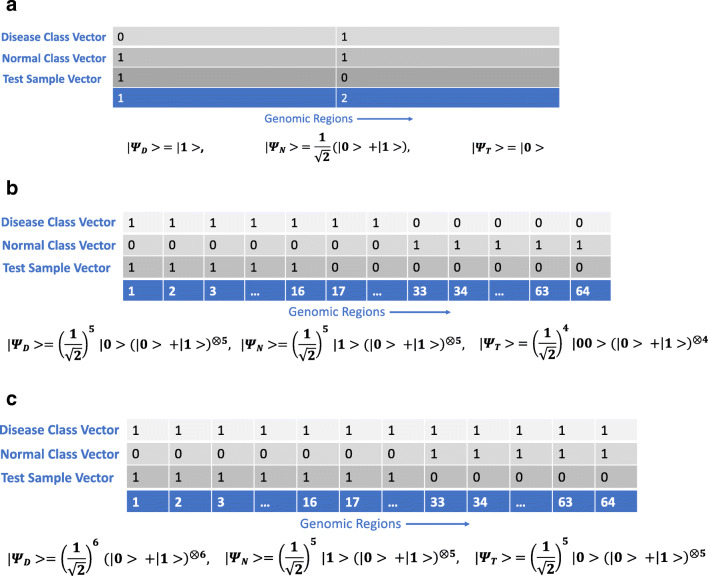
Examples of feature-space vectors of single genomic samples containing different CNV patterns. Each column in the genome represents a feature dimension or physical genomic region. “1” indicates the presence of a CNV in a given region, and “0” indicates its absence. **(a)** shows a case where there are two genomic regions (setup of 5-qubit Example Problem 1), and **(b)** and **(c)** show 64 genomic regions (setup of 14-qubit Example Problem 1 and 2 respectively). Ellipsis points in a dimensional label imply that the vector has the same CNV value for all the unindicated dimensions. Class-vector qubit states for the disease and normal classes, as well the test-vector qubit state, are written with subscripts “D,” “N,” and “T” respectively in the computational basis. They are all shown in the AIP framework for simplicity, though the situation depicted in **(c)** is solved in the manuscript using the SIP framework

The prefactored block states for each individual are vector-summed to yield that individual’s total state vector. An inner product between two individuals’ state vectors would then yield a score quantifying how well the two individual schedules agree. A class is defined generally as a collection of state vectors (henceforth referred to as “training vectors”) satisfying a unique grouping property or pattern. The training vectors of each class are summed to yield its *class vector*. The classifier simultaneously executes an inner product between the test input and each class vector, which is equal to the sum of the inner products between the test vector and each of the class’s training vectors. Based on measurement probabilities, the classifier then classifies the test input into the training class whose class vector yields the highest inner product with the test. In general, the class vector is allowed to be a vector sum of many individuals’ state vectors that belong to the same class (and is prepared based on a classically precomputed sum for the actual circuit). Let us suppose for this example that there were 3 schedules in all, 2 training schedules represented by vectors *A* and *B* each playing the role of a class vector for its respective class, and 1 test input represented by vector *T*. Suppose that there are 8 time blocks and individual *A* has an opening only in the first time block, individual *B* only in the second, and individual *T* in both the second and the third. Thus, |*A*〉 = |000〉, |*B*〉 = |001〉 and $|T\rangle =\frac {1}{\sqrt {2}}(|000\rangle +|001\rangle )$. We immediately see that 〈*T*|*B*〉 > 〈*T*|*A*〉; therefore, the test vector is classified into the class of class vector |*B*〉. This classification is implemented by our inner product classifier. This problem is of course just one example of a broad range of possible applications for this classifier.

The specific motivation for the development of our classifier was a DNA copy number variation (CNV)-based disease classification problem in genomics. The human genome (which is a collection of chromosomes) can be subdivided into smaller regional blocks in chromosomal coordinate space. This is done effectively in the reference genome, which is a consensus of a relatively small number of individual’s genomes and is represented as one large sequence of “A,” “T,” “C,” and/or “G” bases strung together in a line. Each coordinate block of this reference genome is marked for the presence or absence of a CNV as found in a sequenced individual sample genome (any given sample genome itself is not as well studied as the reference and does not have a robust coordinate representation by itself, and hence is “mapped” to the reference coordinate space). For technical details, see for example Trapnell and Salzberg (2009). A CNV in a given regional block indicates a deviation from the number of times (referred to as “copy number”) the genomic string/sequence of that region in the reference genome is expected to occur in the sample genome. The default expected copy number of any region is 2 (there are two copies of each chromosome inherited by every individual from both parents). Copy number variations are associated with a variety of phenotypes and can be strongly correlated with various diseases (lafrate et al. 2004). We looked at CNVs in neuronal cell samples from healthy individuals as well as those affected by Alzheimer’s disease (AD) and developed this classifier as an attempt to classify genomes as healthy or containing AD. This is in fact a very natural specific application for our generic classifier in the regional/spatial domain.

### Binary encoding of feature dimensions into multi-qubit states

Thus, the genome is divided into regional blocks (see Fig. 1) and each block state represents exactly one such region in order. Similar to above, if we subdivided the reference into 64 genomic regions, we would represent them with 6 qubits where a block state of |000000〉 would represent the 1^st^ region, |000001〉 the 2^nd^ region, |111110〉 the 63^rd^ region, and the block state |111111〉 would represent the 64^th^ region. Generally, the state $|f_{n} f_{n-1} {\dots } f_{2} f_{1} \rangle $. represents the $1 + {\sum }_{i=1}^{n} f_{i}2^{i-1}$ region where *f*_*i*_ ∈{0,1}. Thus, we will identify our ordered *n*-qubit computational “basis vectors” (or block states) with basis vectors in feature space in order to encode training and test vectors (referred to as “sample vectors”) similar to Schuld et al. (2017).

### The classification metrics

We employ two classification metrics: the active inner product (AIP) and the symmetric inner product (SIP). First we will list some common features shared by the two metrics. Both metrics have the special merit of being able to handle an arbitrary number of training samples/inputs in the summed class vector form. This is because both are linear: the sum of the inner products between the test vector and each training vector is the same as the inner product between the test vector and the class vector. This means that in one mathematical operation arbitrarily many inner products can be calculated between the test and training vectors and summed together for each class. This is the implementational advantage of our classifier over a raw Hamming distance–based classifier (Ruan et al. 2017; Schuld et al. 2014b), as the Hamming distance is not a linear measure for multiple bit strings in the sense above and would not allow for simultaneous calculation of arbitrarily many mutual distances. This would make implementation on an actual quantum computer a challenge. In fact, SIP (and also AIP under certain conditions applicable to us) is quite precisely the linearized version of Hamming distance (see “Methods” for proofs). Given that the future realization of quantum algorithms on quantum machines is at yet open, speedup may be realized both from a combination of classical and quantum processor calculations or from purely quantum implementations. In the spirit of the former, our circuits encode presummed training data. Due to quantum superposition, the *n*-qubit swap test applied to 2 groups of *n* qubits can simultaneously evaluate the inner product of two *N* = 2^*n*^-dimensional vectors without the need to store each vector component separately (in our presummed formulation, this extends to arbitrarily many *N*-dimensional inner products). The complexity of summing *m* *N*-dimensional vectors classically is *O*(*mN*). Ignoring state preparation, the inner product requires *n* Fredkin gates (18*n* native IBM-Q gates in our implementation of the Fredkin gate) and is therefore $O(\log {N})$ in gate complexity. Thus, assuming the sample vectors are relatively simple and require *O*(1) gates for state preparation or the existence of qRAM which would enable relatively efficient input of sample quantum states, the overall complexity of the combined classical and quantum operations is *O*(*mN*) additions + $O(\log {N})$ gates. The complexity of doing this classically is *O*(*mN*) additions + *O*(*N*) multiplications and additions to calculate the inner product. The relative efficiency of computing the inner product on the quantum computer compared with a classical computer is due to our formulation of encoding each training sample containing genomic attributes as binary-valued features, prior to summing all such samples within a given class. We note that our approach suffers from the general problem of efficiently loading arbitrarily complex classical sample data into a quantum computer (Biamonte et al. 2017; Aaronson 2015; Ciliberto et al. 2018) which some groups are just beginning to address (Cortese 2018).

Furthermore, due to our encoding of presence and absence of CNVs as a state vector coefficient, the sample vectors are easily represented in the computational basis using only unitary (or zero-valued) prefactors for both metrics. In fact, due to this formulation, our inner-product metrics can efficiently apply to a broad category of binary-level problems involving scoring of “matches minus mismatches” or scheduling-like problems as we will shortly see. Moreover, in our framework, there is no theoretical limitation as to the number of dimensions/regions encoded in feature space or the kind of binary-level/CNV-level training data that can be encoded. We note that the difference between the two classification metrics lies in the initial state preparation and not in the inner product implementation itself.

The classes of the training data are represented in our case as disease and normal samples. If the data is separable in some sense, the training vectors for the disease class will have a different regional block pattern for CNV presence compared with those of the normal class. Given a test sample and sufficient separable training data, both the classifiers would classify the test sample into the appropriate category, making disease diagnosis of unknown random samples thus possible. As a technical note, the CNV-level data at this initial stage of the classifier does not allow for distinctions between genomic deletions and duplications.

#### Metric 1: Active inner product

The active inner product (AIP) is defined as the *total* number of times the same region in the test and training vectors for a given class contains a CNV. In the computational basis, the definition is naturally understood to be the inner product of the normalized, sample state vectors. For example, if there were a single sample divided into four genomic regions and only the first 3 had a CNV each, its normalized state vector |*ψ*_1_〉 would be:

1 $$   |{\psi_{1}}\rangle = \frac{1}{\sqrt{3}} \left (|{00}\rangle + |{01}\rangle + |{10}\rangle \right ),  $$

where the state |11〉 is not present due to a coefficient of 0. And if there were a class with two samples including that shown in Eq. 1 and:

2 $$   {|{\psi_{2}}\rangle = \frac{1}{\sqrt{2}} \left (|{00}\rangle + |{11}\rangle \right ),}  $$

corresponding to a CNV in the first and last regions, the normalized class vector, which is the classically pre-computed sum of the two sample vectors, encoded into regions using our binary qubit encoding (Fig. 1) and normalized, is given by:

3 $$   {|{\psi}\rangle = \frac{1}{\sqrt{7}} \left (2 |{00}\rangle + |{01}\rangle + |{10}\rangle + |{11}\rangle \right ).}  $$

We note that non-binary state coefficients in the class vectors are the result of summing ≥ 2 normal or disease samples classically and correspond to ≥ 2 CNVs occurring in a given genomic region across samples. In this case, the normalized class state encodes the occurrence of 2 CNVs in the first region and 1 CNV in the second, third, and fourth regions. In terms of the more general notation introduced in subsection 2.1 that we will use later, Eq. 3 can be written as:

4 $$   {|{\psi}\rangle = \sum\limits_{f1,f2 \in \{0,1\}} c_{f1f2} |{f_{2}f_{1}}\rangle,}    $$

where $c_{00} = \frac {2}{\sqrt {7}}$ and $c_{01} = c_{10} = c_{11} = \frac {1}{\sqrt {7}}$. The inner product is calculated between the test vector and the class vector (the vector sum of the training vectors in each class) simultaneously for the two training classes by overlapping their states via the swap test (Buhrman et al. 2001). The test sample is classified now into its rightful class (implementation details shown shortly). We note that AIP gives preference to “1-matches” over “0-matches.”

Though both AIP and SIP (described next) are well suited for our genomic problem depending upon the context, AIP is the naturally applicable metric for problems like the schedule-matching problem. In fact, it happens to be even better suited than the Hamming distance there. This is because for that particular case one is interested in scoring how well people’s availabilities match, which the AIP renders by summing the total number of 1-matches without any regard for the non-availabilities or 0-matches (unlike Hamming distance). In fact, AIP is a dot product–based kernel classifier broadly applicable to all problems where vector dot products can be used to score similarity between test and train. For our genomic problem, though the Hamming distance is the natural classifier of choice, AIP may be better suited for samples or diseases where the existence of a CNV in the same region in different samples is considered more significant than the absence of a CNV. However, the data that we typically deal with is of such a form that AIP turns out to be equivalent to the Hamming distance as a classification metric, as previously mentioned (see “Methods”).

#### Metric 2: Symmetric inner product

The symmetric inner product (SIP) is defined as the total number of times the same region in the test and training vectors for a given class “match” in terms of CNV presence, minus the number of times they do not match. Broadly applied to any binary-level matching problem, it is the total number of feature matches minus feature mismatches between the test vector and the training set. “Matching” refers to two vectors both having a CNV or not having a CNV in the same region. In the SIP framework, the sample vectors are represented a bit differently in the computational basis compared with the feature basis. For each sample vector, all block states that correspond to genomic regions containing a CNV are assigned a coefficient of 1 whereas regions not containing a CNV are assigned a coefficient of − 1. For example, the sample vector in Eq. 1 would be represented by:

5 $$   {|{\psi_{1}}\rangle} = \frac{1}{2} \left (|{00}\rangle + |{01}\rangle + |{10}\rangle - |{11}\rangle \right )  $$

The rest of the routine proceeds identically to the AIP routine. The reason for the coefficient − 1 in the SIP is that it introduces a penalty for unlike CNV events. Two non-CNV regions or two CNV regions will both contribute + 1 to the inner product, but one CNV and one non-CNV region will contribute − 1 to the inner product to account for mismatches in regional CNV events. SIP is exactly equivalent to Hamming distance as a classification measure (see “Methods”) and is thus naturally well suited to CNV-based genome classification and to all classification problems based on scoring of binary-level matches and mismatches.

### The inner product decision plane

**Fig. 2:**
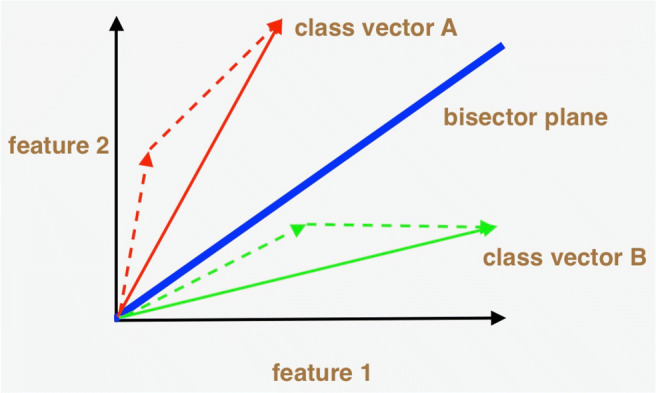
Inner product decision plane in 2 feature dimensions. There are two classes represented in red and green respectively. The class vector for each class is the sum of the class’s respective training vectors, shown with dotted lines. The decision plane, shown in blue, bisects the two class vectors. The test vector will of course be classified according to the side of the decision plane in which it is located. For simplicity, only positive feature coordinates are shown

The decision plane for both the active and the symmetric inner product is the same. As shown in Fig. 2 for 2 feature dimensions, the decision plane is the bisector plane of the two class vectors, as the test vector is classified along with the class vector with which it yields a higher dot product. This of course means that its projection onto that vector is larger than its projection onto the other class vector. For a higher number of feature dimensions, the decision plane is the bisecting hyperplane that is orthogonal to the plane in which the class vectors lie. One advantage of this formulation is that one effectively compares the test vector with arbitrarily many training vectors for each class in one mathematical operation, as these training vectors are summed together into one final class vector for each class. This is possible due to the linearity of the inner product metrics we employ (more in the following section). As an aside, for multiple class vectors, the decision boundary is not a simple hyperplane and is elaborated upon in Online Resource 1.

### The inner product circuit

The overall classical-quantum algorithm and all circuits proceed in the same general way through the following succinct stages:

1. Classically sum *m* disease and normal sample binary vectors each of which contain the CNV profiles (1 for presence or 0 for absence of a CNV in a given genomic region) of each individual across *N* = 2^*n*^ genomic regions to arrive at each feature-space coefficient ($c_{d_{1} {\dots } d_{n}}^{i}$ and $c_{t_{1} {\dots } t_{n}}$ below), which is the number of disease or normal CNVs in a corresponding genomic region divided by a disease or normal state normalization constant. This forms the 2 class vectors, each given by Eq. 7. The complexity of this operation is *O*(*mN*).
2. Encode the feature-space data in the circuit while entangling the class label qubit with the class vectors. The complexity of this operation depends on the complexity of the disease and normal train vectors which can be arbitrarily complex (Biamonte et al. 2017; Aaronson 2015; Ciliberto et al. 2018; Cortese 2018). The circuit is now in state |*ψ*^0^〉.
3. Apply an “*n*-qubit swap” (*n* Fredkin or CSWAP gates), sandwiched between two Hadamard gates on swapper qubit as shown below, directly to *n* paired train and test feature qubits, *d*_1_ and *t*_1_, *d*_2_ and $t_{2},{\dots } d_{n}$ and *t*_*n*_ to prepare a state that will enable calculation of the inner product between the test and entangled class vectors. The complexity of this operation is $O(\log {N})$. The circuit is now in state |*ψ*^*f*^〉.
4. Measure the relevant computational basis probabilities of the swapper and class index qubits that encode the final inner product result. This operation is *O*(1) (it is based on the fixed precision of the state *coefficients*).

The state manipulation routines and circuits can be formulated as generic functions of data parameters (see “Methods”), but we utilize circuit optimizations as suitable to the form of input data in any given problem. We will now look at the different general components/stages of the inner product circuit. In the state equations of this section, we note that the order of the states on the R.H.S. corresponds to the physical order of the qubits that the states occupy on the real hardware (this will be relevant for upcoming swap operations as part of the inner product calculation). In addition, the subscript *following* a state denotes the qubits’ functional role (as opposed to physical qubit position above) and is written in order of the qubits that compose the state vector. The class vector qubit(s) is (are) labelled by the subscript “d,” the class index qubit by “m,” the test vector qubit(s) by “t,” and the swapper qubit by “s.” For example, the two-qubit state |00〉_*md*_ denotes that the left qubit in the state serves to encode the class index and the right one serves to encode the class vector. Furthermore, when |*ψ*〉 is used with a subscript, it represents the state of the circuit when *only* the qubits referred to by the subscript are considered (e.g., |*ψ*〉_*md*_). When |*ψ*〉 is used with a superscript (0 or*f* ), it represents the post-data encoding or final pre-measurement state of the overall circuit respectively.

We now write the generic state for all inner product circuits:

6 $$ {|{\psi}\rangle = \sum\limits_{i=1}^{M} |{i}\rangle_{m} |{d^{i}}\rangle_{d} |{t}\rangle_{t} |{s}\rangle_{s} \frac{1}{\sqrt{M}}, } $$

where |*s*〉_*s*_ is the state of the swapper qubit, |*i*〉 indexes the computational basis state representing the *i*^*th*^ class, |*d*^*i*^〉_*d*_ is the *normalized* class vector state for the *i*^*th*^ class, |*t*〉_*t*_ is the state of the test vector, *M* is the total number of classes, and $\frac {1}{\sqrt {M}}$ is an overall normalization constant stemming from the number of classes. The “s” etc. labels used both in the subscript and state label itself are not redundant as they serve different purposes and are retained for clarity. Now, |*d*^*i*^〉_*d*_ can be specifically written as:

7 $$ {|{d^{i}}\rangle_{d} = \sum\limits_{d_{1} {\dots} d_{n}\in\{0,1\}} c_{d_{1} {\dots} d_{n}}^{i} |{d_{n}d_{n-1} {\dots} d_{2}d_{1}}\rangle_{d},}  $$

where the sum is over all *d*_*j*_ ∈{0,1}, $|{d_{n}d_{n-1} {\dots } d_{2}d_{1}}\rangle _{d}$ represent the training states which encode feature dimensions into qubits as detailed in subsection 2.1, $c_{d_{1} {\dots } d_{n}}^{i}$’s are the class vector coefficients associated with their respective feature states as defined in subsection 2.2.1. As described in subsection 2.2.1, the class states are normalized, 〈*d*^*i*^|*d*^*i*^〉 = 1, yielding ${\sum }_{d_{1} {\dots } d_{n}\in \{0,1\}} |c_{d_{1} {\dots } d_{n}}^{i}|^{2} = 1$. Similarly, the test state |*t*〉_*t*_ can be written as:

8 $$ {|{t}\rangle_{t} = \sum\limits_{t_{1} {\dots} t_{n}\in\{0,1\}} c_{t_{1} {\dots} t_{n}} |{t_{n}t_{n-1} {\dots} t_{2}t_{1}}\rangle_{t},}  $$

where the sum is over all *t*_*j*_ ∈{0,1}, $|{t_{n}t_{n-1} {\dots } t_{2}t_{1}}\rangle _{t}$ represent the test states which encode feature dimensions into qubits and $c_{t_{1} {\dots } t_{n}}$’s are the test vector coefficients associated with their respective feature states. Again, the test state is normalized, 〈*t*|*t*〉 = 1, giving ${\sum }_{t_{1} {\dots } t_{n}\in \{0,1\}} |c_{t_{1} {\dots } t_{n}}^{i}|^{2} = 1$. Thus, $n=\log {N}$ qubits are required in general to index *N* feature dimensions. A key point in the circuit is that the role of an ancilla qubit is subsumed in the class index qubit. Briefly, a useful role for an ancilla qubit is to weight a state containing class qubits associated with the *k*^*th*^ training data point by the distance of that training point to test data when it is measured (Schuld et al. 2017). Thus, measurement of the class qubit in a given class is achieved with a probability equal to the squared distance of the test data to the training data thereby executing quantum based classification (see Schuld et al. (2017) for details). In our case, we only need 1 qubit (to index 2 input classes), which is now directly entangled with the summed training vectors via the sum in Eq. 6.

**Fig. 3:**
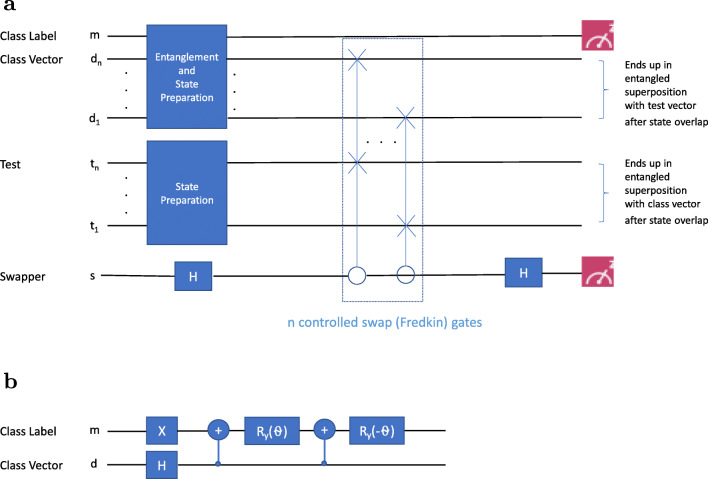
**(a)** The Generic Inner Product Circuit (the initial state of all qubits is |0〉 as usual). The first stage is data-encoding, composed of two state preparation routines executed in parallel. In the second stage, the *n*-qubit swap test is applied on the test and class vector feature qubits with the swapper as control. It consists of 2 Hadamard gates applied to the swapper around the *n*-qubit controlled swap gate (consisting of *n* Fredkin gates as shown), followed by measurement of the swapper. This measurement, along with the class label qubit measurement, yields the inner products. **(b)** As a specific example, gate components inside the “5-qubit entanglement routine” module are shown here

Figure 3 shows the state processing stages (listed at the top of this subsection) clearly for all circuits in general. In the first stage of data encoding, we execute an entanglement routine to encode the training class vector states and entangle them with the class label qubit, as well as a test vector state preparation routine as necessary. These two routines are executed in parallel. All circuits have the same feature space data encoding formulation presented before, which allows for the encoding of 2^*n*^ binary-valued features in *n* qubits. The optimal encoding routines may vary from circuit to circuit and will be specifically presented with the example problems. Notably, while we will maintain a general notation in this section, we develop and assess classifiers that predict one of two classes (*M* = 2), associated with a normal or disease sample in the case of our genomics example. Following data encoding, the equivalent of an “n-qubit swap test” is performed on the test and train (class) vectors, which consists of a Hadamard gate applied on the swapper qubit, followed by the *n*-qubit controlled swap gate, and another Hadamard gate on the swapper before the swapper qubit is measured. In turn, the *n*-qubit controlled swap gate consists of *n* Fredkin (controlled swap) gates which are used to perform *n* controlled swap operations (with each Fredkin gate implemented as in (Smolin and DiVincenzo 1996) by using 2 CNOTs around the Toffoli gate, as presented in Schuld et al. (2017)). These *n* Fredkin gates are applied sequentially to matched train and test qubit feature components *d*_*j*_ and *t*_*j*_ (see Fig. 3(a) and Fig. 5(a)). The *n*-qubit swap test effectively calculates the inner product between the states contained in the qubits it swaps (in our case the test vector and the class vectors). Repeated measurement of the class index qubit in addition to the swapper qubit as shown in Fig. 3(a) reveals the inner products for the 2 classes with the test. From now on, we will simply refer to the *n*-qubit swap test as the “swap test.”

More formally, we represent the state of the circuit after the second stage of data encoding, |*ψ*^0^〉, by substituting Eqs. 7 and 8 into Eq. 6, which shows the form of any generic state. Placing all sums and the normalization constant to the left and using the initial state of |0〉_*s*_ of the swapper qubit:

9 $$   {|{\psi^{0}}\rangle = \frac{1}{\sqrt{M}} \sum\limits_{t_{1} {\dots} t_{n}\in\{0,1\}} \sum\limits_{i=1}^{M} \sum\limits_{d_{1} {\dots} d_{n}\in\{0,1\}} c_{t_{1} {\dots} t_{n}} c_{d_{1} {\dots} d_{n}}^{i} |{\psi^{0}_{i,t_{1} {\dots} t_{n},d_{1} {\dots} d_{n},s}}\rangle,}    $$

where

10 $$   {|{\psi^{0}_{i,t_{1} {\dots} t_{n},d_{1} {\dots} d_{n},s}}\rangle = |{i}\rangle_{m} |{t_{n}t_{n-1} {\dots} t_{2}t_{1}}\rangle_{t} |{d_{n}d_{n-1} {\dots} d_{2}d_{1}}\rangle_{d} |{0}\rangle_{s}.}    $$

Equations 9 and 10 represent the overall state of the circuit shown in Fig. 3 after entanglement and state preparation. The Hadamard gate (*H*_*s*_) is applied to the swapper qubit and *n* Fredkin or CSWAP gates (*CSWAP*^⊗*n*^) are applied to swap the qubits |*d*_1_〉_*d*_ and |*t*_1_〉_*t*_, |*d*_2_〉_*d*_ and |*t*_2_〉_*t*_, $\dots $, |*d*_*n*_〉_*d*_ and |*t*_*n*_〉_*t*_. The effect of these operations on the states of the circuit shown in Eq. 10 is:

11 $$ \begin{array}{@{}rcl@{}}   {CSWAP^{\otimes n} H_{s} |{\psi^{0}_{i,t_{1} {\dots} t_{n},d_{1} {\dots} d_{n},s}}\rangle}   &= \frac{1}{\sqrt{2}} (|{i}\rangle_{m} |{t_{n}t_{n-1} {\dots} t_{2}t_{1}}\rangle_{t} |{d_{n}d_{n-1} {\dots} d_{2}d_{1}}\rangle_{d} |{0}\rangle_{s} \\   &{+ |{i}\rangle_{m} |{d_{n}d_{n-1} {\dots} d_{2}d_{1}}\rangle_{d} |{t_{n}t_{n-1} {\dots} t_{2}t_{1}}\rangle_{t} |{1}\rangle_{s})}   \end{array} $$

Application of another Hadamard gate (*H*_*s*_) to the swapper qubit, use of Eqs. 7–11 yields the overall state of the circuit after the last Hadamard gate operation and before measurement of the swapper and class qubits shown in Fig. 3:

12 $$ \begin{array}{@{}rcl@{}} {|{\psi^{f}}\rangle} &&{=H_{s} CSWAP^{\otimes n} H_{s} |{\psi^{0}}\rangle} \\           &&{=\frac{1}{2} \left( {\sum}_{i=1}^{M} |{i}\rangle_{m} |{d^{i}}\rangle_{d} |{t}\rangle_{t} \cdot \frac{1}{\sqrt{M}} + {\sum}_{i=1}^{M} |{i}\rangle_{m} |{t}\rangle_{t} |{d^{i}}\rangle_{d} \cdot \frac{1}{\sqrt{M}} \right)|{0}\rangle_{s}} \\           &&{+\frac{1}{2} \left( {\sum}_{i=1}^{M} |{i}\rangle_{m} |{d^{i}}\rangle_{d} |{t}\rangle_{t} \cdot \frac{1}{\sqrt{M}} - {\sum}_{i=1}^{M} |{i}\rangle_{m} |{t}\rangle_{t} |{d^{i}}\rangle_{d} \cdot \frac{1}{\sqrt{M}}\right)|{1}\rangle_{s}}           \end{array} $$

Thus, |*ψ*^*f*^〉 is the overall state of the circuit before measurement of the swapper and class qubits shown in Fig. 3. Notably, by performing *n* swaps between matched feature component qubits |*d*_*j*_〉_*d*_ and |*t*_*j*_〉_*t*_, we effectively perform a swap of multi-qubit states |*d*^*i*^〉_*d*_ and |*t*〉_*t*_ with the class index qubit acting as a witness to the swap operation and not participating in the swap even though it is entangled with the class vector qubits. For clarity, the interchange of the *order* of the test and training/class vector states in each term in parentheses signifies the swapping of the states held by respective physical qubits (see examples below for more details). We again note that the state label subscripts (“t,” “d,” etc.) refer to the *function* of the qubit and not to the physical qubit itself and thus remain associated with their respective initial states. Measurements of the swapper and class index qubits are made to arrive at the measurement probability encoding the solution to our problem. To see how this is so, let us calculate some relevant theoretical measurement probabilities. Define $\rho _{\overline {s}k}$ as the probability of measuring the swapper qubit in state $|{s}={\overline {s}}\rangle $ and the class index qubit in state |*i* = *k*〉. After a period of exploring variants of the circuits shown in Figs. 5, 7, 8, we empirically found better agreement between simulated and actual runs on the IBM processors by measuring the swapper qubit in the $\overline {s}=1$ state as compared to $\overline {s}=0$. Thus, we will focus on the measurement probabilities *ρ*_10_ and *ρ*_11_. For detailed analysis of error modes associated with IBM processors, see for example ref. (Sisodia et al. 2017). First, the projection operator projecting the above state onto states corresponding to fixed values of the swapper (*s* = 1) and class index (*i* = *k*), the |*s* = 1〉_*s*_|*i* = *k*〉_*m*_ basis, yields:

13 $$ \begin{array}{@{}rcl@{}} {P(|{s=1}\rangle_{s},|{i=k}\rangle_{m}) |{\psi^{f}}\rangle} &=& \frac{1}{2 \sqrt{M}} \left (\sum\limits_{i=1}^{M} \delta_{ik} |{i}\rangle_{m} |{d^{i}}\rangle_{d} |{t}\rangle_{t} - \sum\limits_{i=1}^{M} \delta_{ik} |{i}\rangle_{m} |{t}\rangle_{t} {|{d^{i}}\rangle_{d}} \right )|{1}\rangle_{s} \\ &=& \frac{1}{2 \sqrt{M}} \left( |{k}\rangle_{m} |{d^{k}}\rangle_{d} |{t}\rangle_{t} - |{k}\rangle_{m} |{t}\rangle_{t} |{d^{k}}\rangle_{d} \right) |{1}\rangle_{s} \end{array} $$

where the Kronecker delta function *δ*_*ik*_ serves to pick out terms corresponding to class *k*. The squared norm of the above is:

14 $$ \begin{array}{@{}rcl@{}} {\rho_{1k}} &=& \frac{1}{4 M} \left( \langle{k}|_{m} \langle{d^{k}}|_{d} \langle{t}|_{t} - \langle{k}|_{m} \langle{t}|_{t} \langle{d^{k}}|_{d} \right) \langle{1}|_{s} \left( |{k}\rangle_{m} |{d^{k}}\rangle_{d} |{t}\rangle_{t} - |{k}\rangle_{m} |{t}\rangle_{t} |{d^{k}}\rangle_{d} \right) |{1}\rangle_{s} \\ &=& \frac{1}{2M} \left( 1 - |A_{k}|^{2} \right) \end{array} $$

where *A*_*k*_ ≡〈*t*|*d*^*k*^〉 is exactly equal to the inner product between the test vector and the class vector of class *k*. Thus, the directly measurable probability *ρ*_1*k*_ is essentially the negative of the squared inner product added to a constant, always a positive number (as is easily verified in Eq. 14) and a monotonically decreasing function of the true inner product of normalized states. All the circuits thus end with a measurement of the probabilities *ρ*_10_ and *ρ*_11_ and our classifier choosing the class *k* that yields the lower measured probability and thus the higher inner product with the test vector.

In terms of data encoding, though specific routines for encoding data will be employed in our circuits, the kind of data that can be entered in principle is a little less limited (see “Methods”). As mentioned above, we do not attempt to solve the general data input problem associated with quantum computing (Biamonte et al. 2017; Aaronson 2015; Ciliberto et al. 2018; Cortese 2018). Nevertheless, we do provide a recipe in “Methods” for encoding data exhibiting certain properties/symmetries. However, circuits encoding non-trivial data may have extremely large gate depth in large part due to the limitations of CNOT connectivity in currently available quantum processors. Therefore, for relevance and clarity, we will present problems involving simplified data that can be input feasibly into IBM machines. These solutions themselves require non-trivial gate-depth at times as they need several swap operations.

**Fig. 4:**
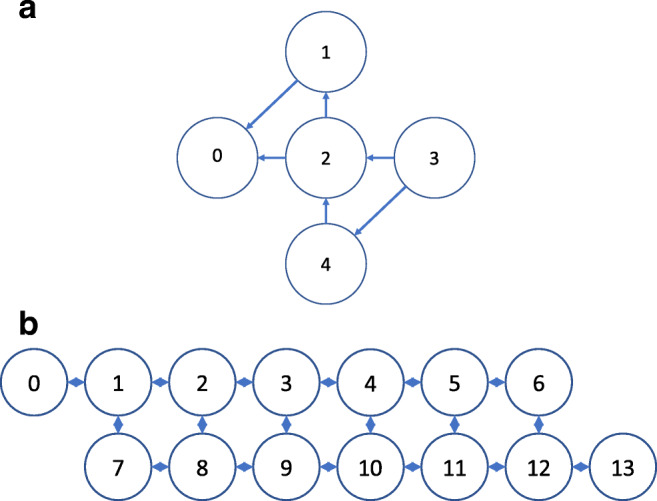
Underlying CNOT connectivity constraints of **(a)** ibmqx2 and **(b)** ibmq_16_melbourne at the time of circuit implementation. Qubit numbers are circled and CNOT connections shown via directional arrows. A unidirectional arrow specifies the constraint that the originating qubit can only act as control and the destination qubit only as the target of a CNOT operation. A symmetric diamond shape for connections in **(b)** indicates that either qubit can serve as control or target. These qubit numbers correspond exactly to the qubit numbers shown for the real IBM processors in Figs. 5 and Online Resource 2

We now present our circuits with their respective example problems. We will first describe the 5-qubit circuit and then the 14-qubit version. For each, problems are solved using the simulator as well as the real processor using the AIP and/or SIP framework as applicable. 1 For each case, we will first introduce the generic circuit and state notation, and then present specific example problems. The underlying connectivity constraints of the backends used at the time of circuit implementation are shown in Fig. 4.

### Five-qubit generic circuit

AIP problems involving an artificial genome containing two regional blocks can be solved with the 5-qubit circuit. The SIP framework does not have much relevance or power here due to the small feature space. Only 1 qubit was used to encode the class vector (“d”), which spans the two genomic regions. The other 3 qubits were taken by the class index (“m”), test vector (“t”), which also resides in 2-dimensional genomic feature space, and the swapper qubit (“s”).

As we have two classes to index, class-index-qubit state |0〉_*m*_ represents class 0 (disease) and |1〉_*m*_ represents class 1 (normal). For all 5-qubit circuits we will present, this class index qubit is first entangled with the training qubit using a particular entanglement routine so as to separate the training vectors for each class (see Fig. 3(b)). Our 5-qubit “generic entanglement routine” (a similar but functionally different module is presented in Schuld et al. (2017)) allows one to prepare a relatively broad class of entangled two-qubit states involving the class label and class vector qubits. In these states, the disease class state always has a CNV in the second region only corresponding to pre-summing all disease samples having a CNV in the second region and generating a normalized disease class state; hence, |*d*^0^〉_*d*_ = |1〉_*d*_. The normal class state contains CNVs in both regions, which corresponds to pre-summing multiple samples containing CNVs in either or both regions and generating a normalized normal class state. Using the form of Eq. 7, the normal class state can be written as $|{d^{1}}\rangle _{d} = {c_{0}^{1}} |{0}\rangle _{d} + {c_{1}^{1}} |{1}\rangle _{d}$ where $|{c_{0}^{1}}|^{2} + |{c_{1}^{1}}|^{2} = 1$. Without loss of generality, we set ${c_{0}^{1}} = \sin \limits {\theta }$ and ${c_{1}^{1}} = \cos \limits {\theta }$ (*𝜃* here happens to be precisely the rotation angle shown in Fig. 3(b)). 2 This state of the class and train qubits, |*ψ*^0^〉_*md*_, is generated after action of the 6 gates shown in Fig. 3(b) on the state |00〉_*md*_. This generic post-data encoding state for the 5-qubit inner product circuits reads:

15 $$   {|{\psi^{0}}\rangle_{md} = \frac{1}{\sqrt{2}} (|{01}\rangle_{md} + \sin{\theta}|{10}\rangle_{md} + \cos{\theta}|{11}\rangle_{md})}   \label {5qGenState} $$

where the normalization $\sqrt {2}$ corresponds to *M* = 2 classes (as shown in Eq. 6). This state refers to the general situation where class 0 (disease) CNVs lie wholly in the second genomic region and the number of class 1 (normal) CNVs in the first genomic region is scaled by a factor of $\tan {\theta }$ relative to the number in the second genomic region (please see discussion on raw normalization/scaling factor in “Methods”). As a specific example, the state $|{\psi ^{0}}\rangle _{md} = \frac {1}{\sqrt {2}}(|{01}\rangle _{md} + |{10}\rangle _{md})$ denotes the simple case that in class 0 (disease) there is a training state with a CNV only in the second of two regions and in class 1 (normal) there is one CNV in the first region.

#### Example problem 1: AIP on 2-block genome

We will solve a very simple problem with the 5-qubit circuit now in the AIP framework on the simulator as well as the ibmqx2 backend. The underlying connectivity constraints of ibmqx2 at the time of circuit implementation are shown in Fig. 4(a). In this example, the normal class vector (class 1) has one training vector that contains a CNV in each of the 2 total genomic regions. The disease class vector (class 0) has only 1 CNV, present in the second region. The test vector also has only 1 CNV, present in the first region (see Fig. 1(a)). Thus, the test sample should be classified as normal. In the data-encoding stage, the 5-qubit generic entanglement routine is used with $\theta = \frac {\pi }{4}$, which when used in Eq. 15 yields the post-entanglement routine state for this circuit: $|{\psi ^{0}}\rangle _{md}=\frac {1}{\sqrt {2}}|{01}\rangle _{md} + \frac {1}{2} (|{10}\rangle _{md} + |{11}\rangle _{md})$. The test vector is already in the desired form and is not acted upon in the data-encoding stage.

**Fig. 5:**
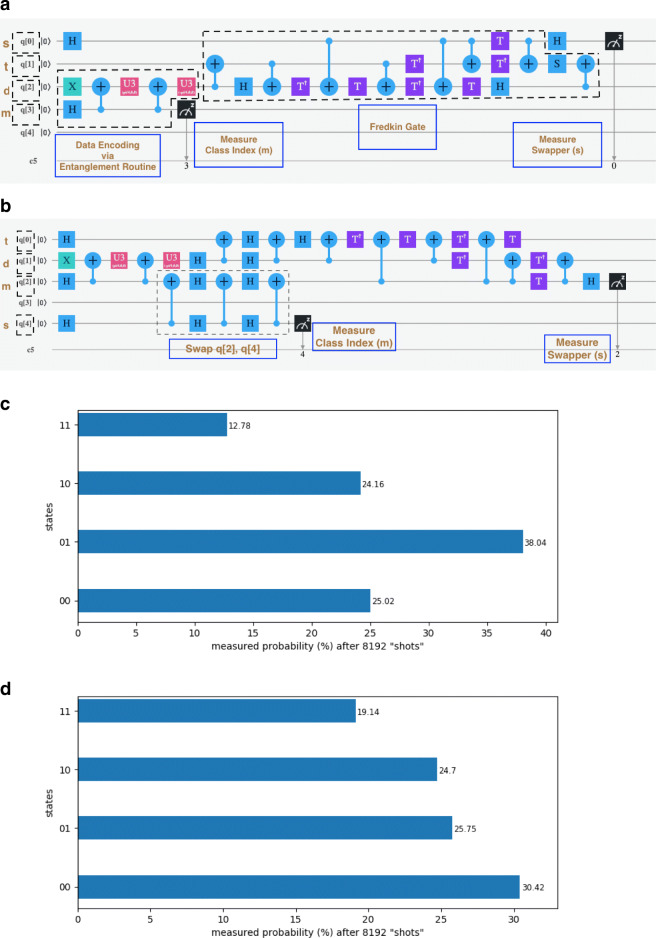
5-qubit example 1 as drawn on IBM Quantum Experience Composer (IBM-Q-team 2019c). We show **(a)** the unconstrained simulator circuit with labelled submodules including the complete Fredkin gate, **(b)** the ibmqx2 circuit with labels including the architectural qubit-swap, **(c)** measurement probabilities of the ibmqx2 circuit on simulator, and **(d)** measurement probabilities of same on ibmqx2. In **(a)**, the predefined functional qubit labels are used to clarify the roles of the 4 qubits used (“s” for swapper, “t” for test, “m” for class index, and “d” for class vector). The swap test consists of “H” on swapper, Fredkin/controlled swap gate with swapper as control and “t,” “d” qubits as targets, respectively, and again “H” on swapper. In **(b)**, the 2-qubit swap gate, a necessity for ibmqx2 connectivity constraints, swaps the qubits for the class index and swapper qubits. The functional qubit labels of course apply only until relevant qubits are swapped. In **(b)** and **(c)**, the measured values of only the swapper and class index qubits are shown for clarity in that order

At this point, the test and swapper qubit states are, formally, |*t*〉_*t*_ = |0〉_*t*_ and |*s*〉_*s*_ = |0〉_*s*_, yielding the initial state:

16 $$ \begin{array}{@{}rcl@{}} {|{\psi^{0}}\rangle = |{0}\rangle_{s} |{0}\rangle_{t} \left( \frac{1}{\sqrt{2}}|{1}\rangle_{d}|{0}\rangle_{m} + \frac{1}{2}(|{0}\rangle_{d} + |{1}\rangle_{d}) |{1}\rangle_{m} \right),} \end{array} $$

in which the qubit order is that of the simulator circuit shown in Fig. 5(a). The second stage of the circuit is the swap test applied on the training and test qubits with the swapper qubit as control, which evaluates the inner product between the class vector and test vector qubit states (see Fig. 5). This inner product value is given by the measurement probabilities of the state of the “s” and “m” qubits as measured in the computational basis, which leads us to the last phase of the computation. 8192 “shots” are taken, and *ρ*_10_ and *ρ*_11_ are measured and plotted on a histogram (for simplicity, no additional subscripts shall be used for these quantities for simulated or real runs; it should be clear from the context whether they correspond to a theoretical calculation, simulation or real run). As a prior, the theoretical values for the inner product are set by Eq. 14, yielding $\rho _{10} = \frac {1}{4}$ and $\rho _{11} = \frac {1}{8}$, showing that the test vector yields a higher inner product with class 1 and should be classified as such.

This problem is solved first on the IBM simulator on a version of the circuit not limited by architectural constraints, then on the simulator using a circuit appropriate for the ibmqx2 architecture, and finally using the same circuit on the ibmqx2 processor itself. The latter circuit along with the histogram of measured probabilities for the simulation and the real execution is shown in Fig. 5. The unconstrained simulation circuit is shown in Online Resource 2. The real and simulated circuits differ only in the order of the qubits used to encode the data, and in the swap operations required to satisfy the constraints of the real processor. In Table 1, we compare probability values from the theoretical calculation, “measurements” from the architecturally constrained simulation, and measurements from the real execution for the various (5-qubit and 14-qubit) circuit examples. The simulation Fig. 5(c) yields $\frac {\rho _{11}}{\rho _{10}} = \frac {12.78}{24.16} = 0.53$, while ibmqx2 in Fig. 5(d) gives $\frac {\rho _{11}}{\rho _{10}} = \frac {19.14}{24.7} = 0.77$. As expected, the simulation probability is almost identical with the theoretical one for this example and classifies the test sample correctly as normal. The real measured probability, though 45*%* away from the expected value, classifies the sample correctly.

**Table 1:** The ratio $\frac {\rho _{11}}{\rho _{10}}$

Problem	Theory	Simulation	Real
5-Qubit example 1	$\frac {1}{2}$	0.53	0.77
14-Qubit example 1	2	1.9	0.26
14-Qubit example 2	0	0.0	n/a

#### ibmqx2 performance across test state classifiability

**Fig. 6:**
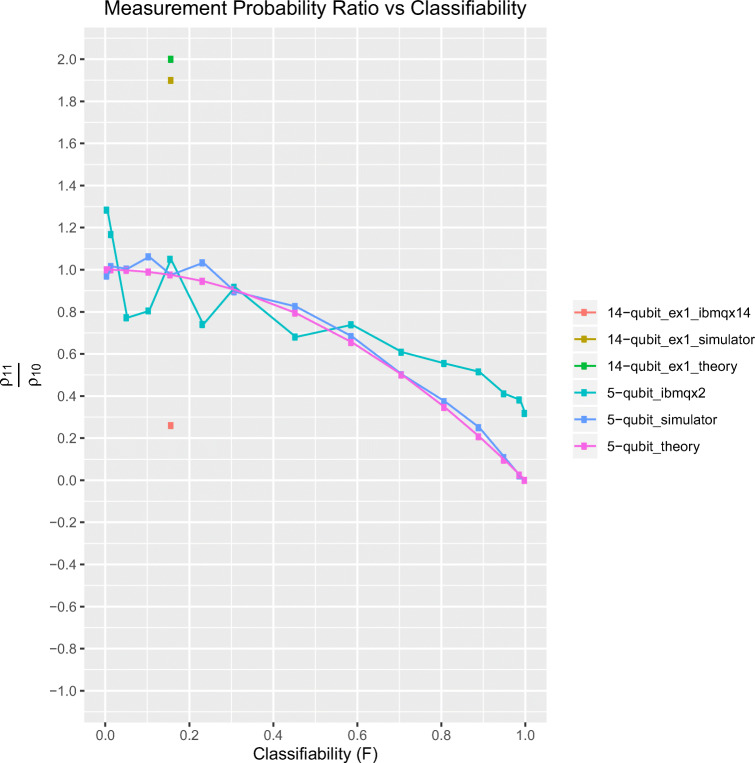
Plot of measurement probability ratio $\frac {\rho _{11}}{\rho _{10}}$ vs classifiability F=|*A*_1_ − *A*_0_| for the 5-qubit generic circuit simulation and ibmqx2 run as well as for the simulation and ibmq_16_melbourne run of the single point experiment, “AIP on 64-block genome.” The 5-qubit circuit plot was created by varying the *𝜃* parameter ($0 < \theta \leq \frac {\pi }{2}$), effectively plotting over the range 0 < *F* ≤ 1 (as $F=\sin \limits {\theta }$). The first point is plotted at $\theta = \frac {\pi }{600}$, almost at the unclassifiable point of *F* = 0. All y-values below 1 for this circuit indicate that the test data was correctly classified. All test cases corresponding to $\theta > \frac {\pi }{20}$, or *F* > .157, were correctly classified by ibmqx2. For the 14-qubit experiment, the single instances of the simulation and ibmq_16_melbourne run are plotted to serve as comparison. Correct classification corresponds to y-values above 1 for this circuit. It is immediately obvious that the ibmq_16_melbourne run performs (not surprisingly) far worse than any of the 5-qubit experiments including incorrectly classifying the test state

In order to assess the efficacy of our circuit and the overall circuit fidelity of ibmqx2, we executed the 5-qubit generic circuit on the simulator and on ibmqx2 to plot performance over the full range of a *classifiability* parameter, *F*. We define classifiability generally as the absolute value of the difference in the theoretical inner product between the test and normal class and that between the test and disease class: *F* = |〈*t*|*d*^1^〉−〈*t*|*d*^0^〉| = |*A*_1_ − *A*_0_|. Because the state coefficients in the AIP framework are always positive, the inner products 〈*t*|*d*^*i*^〉≥ 0 for *i* ∈{0,1}. Thus, *F* ranges from 0 (completely unclassifiable), corresponding to the training state being equidistant to both the normal and disease class states, to 1 (completely classifiable), corresponding to the test state being identical to either the disease or normal class state and orthogonal to the normal or disease class state, respectively. The test input used was again |*t*〉_*t*_ = |0〉_*t*_. The quantity plotted on the y-axis is our usual classification metric $\frac {\rho _{11}}{\rho _{10}}$, the “measurement probability ratio” (MPR). Three curves are plotted for MPR corresponding to theory, simulation and the real run. We can see from Eq. 15 that *A*_0_ = 〈*t*|*d*^0^〉 = 〈0|1〉 = 0 and $A_{1}= \langle {t}|{d^{1}}\rangle = \sin \limits {\theta }$; thus, classifiability reduces to $F=\sin \limits {\theta }$. From Eq. 14, values of MPR < 1 correspond to correctly classified test data. As can be seen from Eq. 15, *𝜃* ≈ 0 corresponds to all normal sample CNVs being in the second region, and increasing *𝜃* yields higher relative numbers of normal CNVs in the first region with $\theta = \frac {\pi }{4}$ giving an equal number of normal CNVs in both regions and $\theta = \frac {\pi }{2}$ corresponding to normal CNVs in the first region only. In the plot shown in Fig. 6, *F* ranges in discrete steps from values corresponding to *𝜃* ≈ 0 3 (almost unclassifiable as *F* ≈ 0) to $\theta =\frac {\pi }{2}$. 4096 “shots” were taken to produce each data point. It is notable that for $\theta > \frac {\pi }{20}$, or *F* > 0.157, all test cases were correctly classified by ibmqx2. It is also noteworthy that the real run MPR values oscillate about the theoretical and simulated values with the oscillation amplitude dampening with increasing *F* until an increasing degradation in ibmqx2 performance compared with theory and simulation for *F* ≥ 0.55, while still classifying samples correctly. In addition to the 5-qubit generic circuit experiments, a single point corresponding to the 14-qubit example problem 1 is also plotted on the same graph (see “Example problem 1: AIP on 64-block genome” in the current section).

### Fourteen-qubit generic circuit

With the 14-qubit circuit, we can solve problems involving a simple genome containing up to 2^6^ = 64 regional blocks (with some optimizations this can be extended if there are any CNV-absent regions—see “Zero-coefficient exclusion” in “Methods”). Using the same notation scheme as for the 5-qubit circuit, 1 qubit was used to encode the class index, 6 qubits were used for the class vectors (labelled individually by $d_{1},d_{2} {\dots } d_{6}$ in increasing order of bit place value, or simply by “*d*” collectively), 6 qubits for the test vector ($t_{1},t_{2} {\dots } t_{6}$ individually or “*t*” collectively) and 1 for the swapper qubit (“s”). We note that the dual use of the labels *s*, *d*_1_ etc. as both functional qubit indices and state labels is quite natural and unambiguous, e.g., |*s*〉_*s*_ or ${\phantom {0}}|{d_{1}}\rangle _{d_{1}}$.

#### Example problem 1: AIP on 64-block genome

We solve this problem on the 14-qubit simulator as well as the ibmq_16_melbourne backend using the Qiskit software for circuit assembly (Abraham et al. 2019). The underlying connectivity constraints of the backend at the time of circuit implementation are shown in Fig. 4(b). For this problem, a single CNV is present in each of the first 32 regions (1 − 32) only in the class vector for class 0 (disease), and each of the last 32 regions (33 − 64) only in the class vector for class 1 (normal). The test vector represents a test sample that contains a CNV in each of regions 1 − 16 only (see Fig. 1(b)). As a practical matter, the class vectors of this (and the following) example would be composed of multiple sample vectors to have this particular form in our context. The test sample here should be classified into class 0 as a disease sample.

**Fig. 7:**
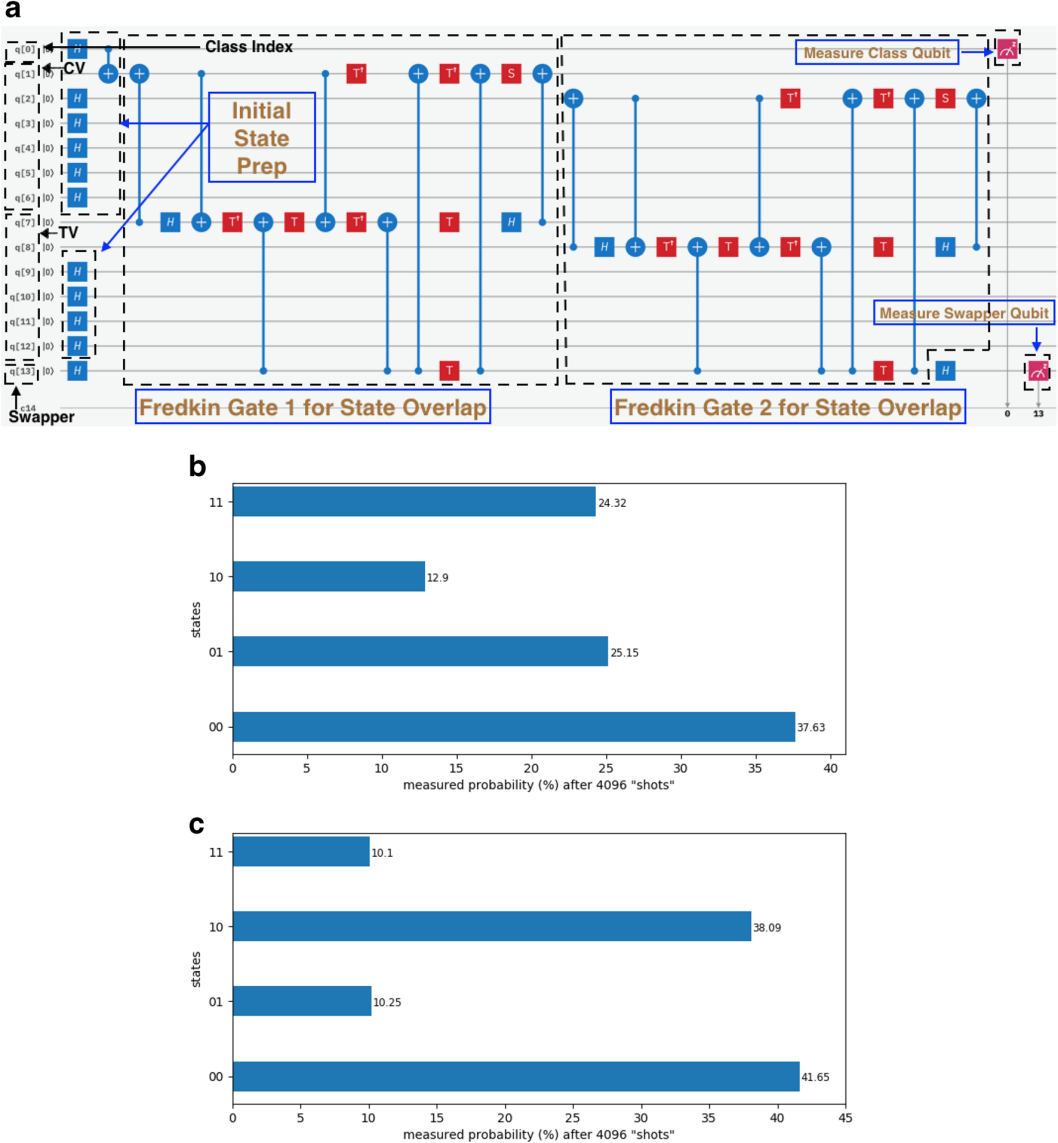
14-qubit example problem 1 as drawn on IBM Quantum Experience Composer (IBM-Q-team 2019c). We show **(a)** the simulator circuit, **(b)** measurement probabilities on simulator, and **(c)** measurement probabilities on ibmq_16_melbourne. The circuit adapted to satisfy ibmq_16_melbourne connectivity constraints is too cumbersome to show here and is shown in Online Resource 3. The measurement probabilities shown pertain to the adapted version. “CV” refers to “class vector” and “TV” to “test vector.” In the histograms, only the measured values of the swapper and class index qubit states are shown for clarity in that order

As previously, |0〉_*m*_ is the first computational basis state representing class 0 and |1〉_*m*_ the second representing class 1. As a note, in the simulated version of the problem, the first qubit (*q*0) is used for class index, *q*1 − *q*6 for class vectors, *q*7 − *q*12 for the test vector (both in decreasing order of place value of bits, *d*_6_ − *d*_1_ and *t*_6_ − *t*_1_, respectively), and *q*13 for the swapper qubit. This changes in the real-processor version as shown in Online Resource 3 for ease of required swap operations. The class index qubit is first entangled with the 6 training qubits as follows (see also Fig. 7). A Hadamard gate is applied to *m*, followed by a CNOT gate with *m* in control and *d*_6_ as target. This puts the first 7 qubits in the following superposition, effectively entangling the class index qubit with the 6 training qubits:

17 $$   |{\psi}\rangle_{md} = \frac{1}{\sqrt{2}}\left( |{0}\rangle_{m} |000000\rangle_{d} + |{1}\rangle_{m} |{1000000}\rangle_{d} \right)    $$

To encode our data optimally, a Hadamard gate is applied to *d*_5_ − *d*_1_ each and *not* to *d*_6_. This puts |*ψ*〉_*md*_ in the state:

18 $$   {|{\psi}\rangle_{md} = \left( \frac{1}{\sqrt{2}}\right)^{6} \left( |{0}\rangle_{m} |{0}\rangle_{d_{6}}(|{0}\rangle+|{1}\rangle)^{\otimes 5}_{d_{5}-d_{1}} + |{1}\rangle_{m} |{1}\rangle_{d_{6}} (|{0}\rangle+|{1}\rangle)^{\otimes 5}_{d_{5}-d_{1}} \right)}    $$

where the subscripted tensor product $(|{0}\rangle +|{1}\rangle )^{\otimes 5}_{d_{5}-d_{1}} \equiv (|{0}\rangle +|{1}\rangle )_{d_{5}}(|{0}\rangle +|{1}\rangle )_{d_{4}}(|{0}\rangle +|{1}\rangle )_{d_{3}}(|{0}\rangle +|{1}\rangle )_{d_{2}}(|{0}\rangle +|{1}\rangle )_{d_{1}}$ etc. This state exactly represents the CNV configuration in the problem definition per the AIP framework (see Fig. 1).

At this point, the qubit states for the other qubits are as follows: |*t*〉 = |000000〉_*t*_ and |*s*〉 = |0〉_*s*_. Now, using the same technique as above to achieve the desired test vector, a Hadamard gate is applied to *t*4 − *t*1 each, yielding the pre-swap test state:

19 $$ \begin{array}{@{}rcl@{}} |{\psi^{0}}\rangle = \left( \frac{1}{\sqrt{2}}\right)^{10} \left( |{0}\rangle_{m} |{0}\rangle_{d_{6}}(|{0}\rangle+|{1}\rangle)^{\otimes 5}_{d_{5}-d_{1}} + |{1}\rangle_{m} |{1}\rangle_{d_{6}} (|{0}\rangle+|{1}\rangle)^{\otimes 5}_{d_{5}-d_{1}} \right)\\ |{00}\rangle_{t_{6}-t_{5}}(|{0}\rangle+|{1}\rangle)^{\otimes 4}_{t_{4}-t_{1}} |{0}\rangle_{s}. \end{array} $$

In this case, only two Fredkin gates are required as shown in Fig. 7(a) in order to swap the pairs |*d*_6_〉_*d*_, |*t*_6_〉_*t*_ and |*d*_5_〉_*d*_, |*t*_5_〉_*t*_ given that the remaining feature qubit states are the same. The swap test is executed as usual to encapsulate the inner product into the measurement probabilities of the states of the “s” and “m” qubits in the computational basis; 8192 “shots” are taken, and *ρ*_10_ and *ρ*_11_ are measured and plotted on a histogram. In this case, the theoretical measurement probabilities from Eq. 14 are $\rho _{10} = \frac {1}{8}$ and $\rho _{11} = \frac {1}{4}$, showing that the test vector yields a higher inner product with class 0 and should be classified as a disease sample. As before, the problem is first straightforwardly solved on the simulator, then on the simulator using an architecturally constrained circuit and finally on the ibmq_16_melbourne processor. The measured results along with the circuit are shown in Fig. 7. Again, in Table 1, the theoretical probabilities, the architecturally simulated probabilities, and the real measured probabilities can be seen. The simulated probability is very close to the theoretical value as expected. However, due to current limitations in hardware, measurements from the real processor yielded a ratio $\frac {\rho _{11}}{\rho _{10}} = 0.26$ that significantly departed from the theoretical value of 2 and did not classify the sample correctly. Notably, the introduction of a second Fredkin or CSWAP gate (composed of 18 ibmq_16_melbourne gates) degraded performance significantly compared with the 5-qubit system in which one Fredkin or CSWAP gate was implemented. While reduced performance is expected, this single ibmq_16_melbourne experiment highlights the current limitations of implementing even more than one Fredkin or CSWAP operation with current hardware. This reduced performance is represented in Fig. 6 via comparison with the expected theoretical and simulation values for this example problem.

Again, one can see in the circuit that the swap test was only applied to some of the test and class vector qubits and not to all. This is an optimization specific to this circuit as mentioned in “Inner product circuits” employing the principle of summing coefficient products of only like-valued qubits to calculate the inner product (please refer to “Optimization techniques in methods”).

#### Example problem 2: SIP on 64-block genome

This problem was solved on the simulator. As shown in Fig. 1(c), for this problem a single CNV is present in each of the 64 regions in the class vector for class 0 (disease), and each of the last 32 regions (33 − 64) only in the class vector for class 1 (normal). The test vector represents a test sample that contains a CNV in each of regions 1 − 32. Thus, the test sample should be classified into class 0. As mentioned in “Methods,” only the square of the SIP, i.e., square of the difference between matches and mismatches and not the sign of the inner product, is actually measurable (see “Methods” for work attempting to address this issue indirectly). However, this is no problem for the data we used to guide the development of our classification metrics (van den Bos et al. 2016) (pioneering single-cell whole genome sequencing data for the context of our quantum classifier attempting to classify neuronal disease based on CNVs in samples, hereupon referred to as “contextual data”), where the number of matches always exceeds the number of mismatches (see SIP in “Methods”). This example is fabricated differently from the actual data to highlight certain features of the circuit 4 and here we will posit that the number of matches is not expected to exceed the number of mismatches, as shown in the genomic setup in Fig. 1(c). This means that the measured inner product between the test and class 1 vector (stemming from an SIP of absolute value 64, due to 0 matches and 64 mismatches) is expected to be higher than the inner product between the test and class 0 vector (stemming from an SIP of 0, due to 32 matches and 32 mismatches).

**Fig. 8:**
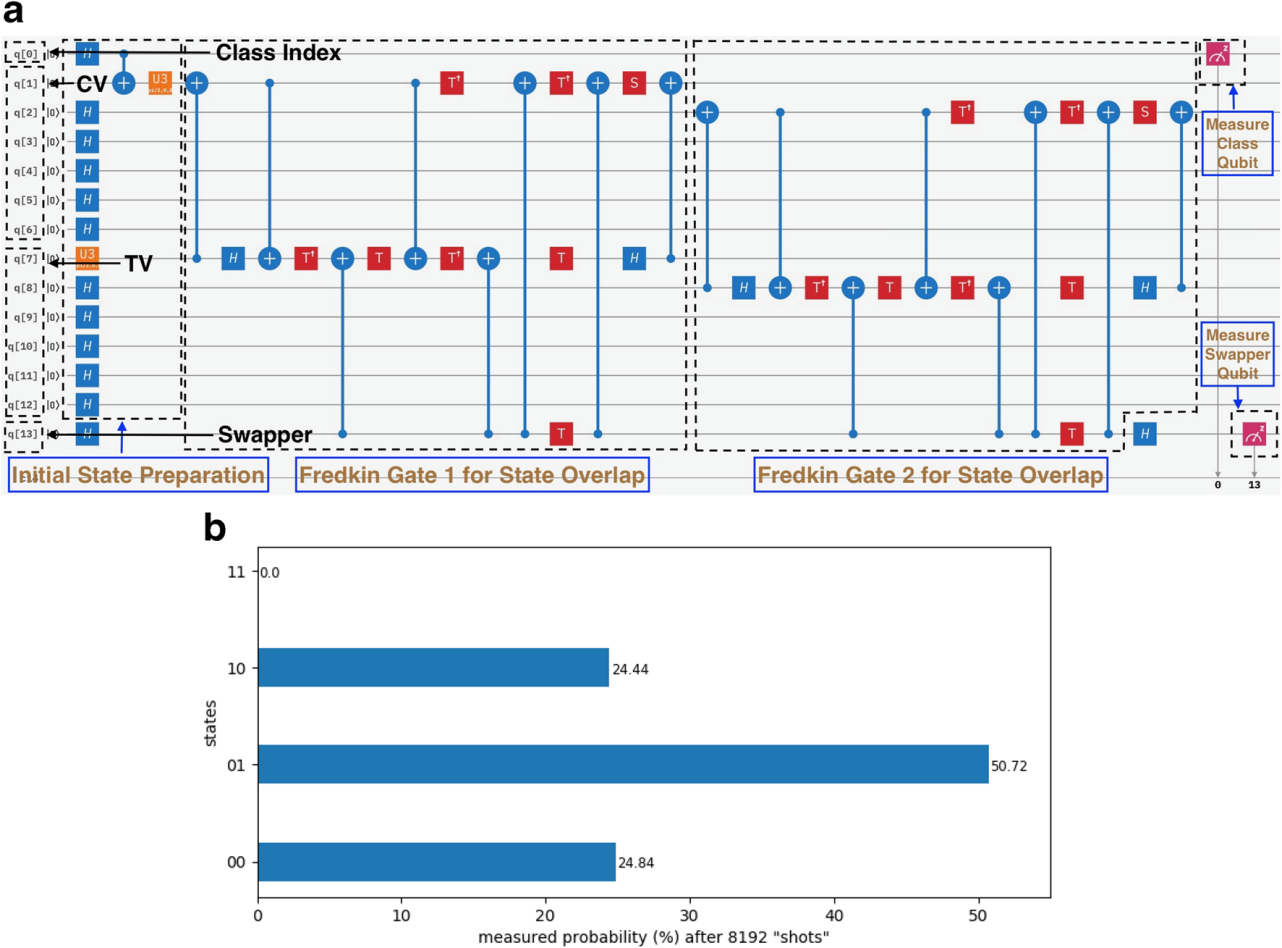
14-qubit example problem 2 as drawn on IBM Quantum Experience Composer (IBM-Q-team 2019c). We show **(a)** the circuit and **(b)** measurement probabilities on simulator. “CV” refers to “class vector” and “TV” to “test vector.” In the histograms, only the measured values of the swapper and class index qubit states are shown for clarity in that order

In the same way as in the above example (see Fig. 8), we create the state:

20 $$ \begin{array}{@{}rcl@{}}   |{\psi}\rangle_{md} = \frac{1}{\sqrt{2}}\left( |{0}\rangle_{m} |000000\rangle_{d} + |{1}\rangle_{m} |{1000000}\rangle_{d} \right) \end{array} $$

and apply $R_{y} \left (\frac {\pi }{2}\right )$ to *d*_6_, and $R_{y} \left (-\frac {\pi }{2} \right )$ to *t*_6_, to arrive at the full state:

21 $$ \begin{array}{@{}rcl@{}} |{\psi^{0}}\rangle = \left( \frac{1}{\sqrt{2}}\right)^{13} \left( |{0}\rangle_{m} (|{0}\rangle+|{1}\rangle)^{\otimes 6}_{d_{6}-d_{1}} + |{1}\rangle_{m} (|{1}\rangle-|{0}\rangle)_{d_{6}} (|{0}\rangle+|{1}\rangle)^{\otimes 5}_{d_{5}-d_{1}} \right)\\ (|{0}\rangle - |{1}\rangle)_{t_{6}} (|{0}\rangle+|{1}\rangle)^{\otimes 5}_{t_{5}-t_{1}} |{0}\rangle_{s}. \end{array} $$

The swap test was applied and the simulated probabilities measured as usual. We compare them with their theoretical counterparts in Table 1 and see that they are extremely close as expected. The test sample was classified into class 0, which yielded the lower inner product, due to the precondition indicating fewer mismatches with class 0. This example was not run on the real processor simply because, given current hardware limitations, it would add no value to our results. We have already demonstrated how a very similar design can be mounted on ibmq_16_melbourne.

### Summary

We have thus presented several example problems on both the 5-qubit and 14-qubit versions of IBM processors. We see that the 5-qubit circuit results are much more aligned with their theory and simulation counterparts than those of the 14-qubit circuit. The deviation in the latter is due to limitations associated with circuit implementation and execution. In principle, and especially in the presence of qRAM to address the quantum data input problem, the circuits are an improvement over their classical counterparts due to the following main features:

1. They provide a feature space mapping for bit vectors by using them as coefficients of computational basis states, encoding 2^*n*^ binary features in *n* qubits.
2. The classifier is designed as a linearized version of a Hamming-distance–based classifier, allowing for the use of presummed input training data into class vectors. The inner product between this data and the test vector calculated using the *n*-qubit swap test effectively encodes a summed inner product with arbitrarily many training inputs for the 2 classes.
3. We combine the training data for the 2 classes (extendable to any number *m*), each containing 2^*n*^ features, into *n* qubits (as opposed to *m* ⋅ *n*), by entangling training vectors with the class index qubit.
4. Finally, the quintessential quantum advantage of manipulating 2^*n*^ basis states and coefficients in superposition simultaneously in an *n*-qubit machine is fully realized in this design, as essentially *n* Fredkin gates calculate the sum of the product of 2^*n*^ dimensional vector components. The *execution* advantage is $O(\log {N})$ vs 0(*N*) in a classical machine.

To reiterate the last point in our context, the class vector components can have arbitrarily high precision in principle (set by how precise the physical rotation angles are in the hardware) and yet the product of each pair of test-class vector components is encoded into the probability amplitude via merely one Fredkin gate. The number of native IBM-Q gates used in implementing 1 Fredkin gate is a constant (18 in the implementation we used). Of course, the number of measurements required to establish confidence in the result will increase with vector precision.

## Methods

### Hamming distance and inner product equivalence

All individual sample vectors (training or test) have binary-valued components (indicating in our context the presence or absence of a CNV in the regional dimensions of feature space). For what follows, we define the “bit-string-equivalent” (BSE) for a binary-valued sample vector such that the vector’s *i*^*th*^ component is simply treated as the value of the *i*^*th*^ bit of its BSE. Then, the Hamming distance between two binary-valued sample vectors in feature space is naturally defined to be the Hamming distance between their respective BSEs. We will work out below the equivalence between the Hamming distance and the SIP/AIP of two sample vectors in the sense of their being equivalent classification measures. We will also show how SIP and AIP are linear in the sample vectors while the Hamming distance is not.

#### SIP

We will first prove the linear behavior of SIP in terms of sample vectors and show how it is implemented as a veritable classifier in a quantum machine. Then, the proof of its equivalence with Hamming distance will follow. An *F*-dimensional class vector *in feature space* takes the general form $C=\frac {1}{N_{C}}{\sum }_{f=1}^{F} c_{f} \vec {f}$, where $\vec {f}$ is the indexed standard basis vector in *F*-dimensional feature space, *c*_*f*_’s are un-normalized vector components or regional prefactors of *C*, and *N*_*C*_ is an overall normalization constant. Suppose *C* is composed of an arbitrary number of training vectors, which in analogous notation take the form $D=\frac {1}{N_{D}}{\sum }_{f=1}^{F} {a_{f}^{D}} \vec {f}$, where we note that *D* serves as a training index and ${a_{f}^{D}}$ is a binary regional prefactor in feature space for the *D*^*th*^ training vector. Suppose also that we have a test sample given by the vector $B=\frac {1}{N_{B}}{\sum }_{f=1}^{F} b_{f} \vec {f}$ with notation exactly analogous to *D*. The Hamming distance between *D* and *B* is now by definition:

22 $$ H(B,D)=\sum\limits_{f=1}^{F}(1-\delta_{{a_{f}^{D}} b_{f}})  $$

where $\delta _{{a_{f}^{D}} b_{f}}$ is the standard Kronecker delta function applied to ${a_{f}^{D}}$ and *b*_*f*_. We are interested in minimizing the Hamming distance between sample vectors for optimal classification, which is equivalent to maximizing the reverse Hamming distance or the quantity − *H*.

For clarity, we will focus on one training class only for this proof and generalize at the end. Let us first define *S*_11_(*B*,*D*) as the total number of times corresponding vector components/prefactors of *D* and the test vector *B* are both 1, i.e.:

23 $$ S_{11}(B,D)= \sum\limits_{f=1}^{F} \delta_{{a_{f}^{D}} 1} \delta_{b_{f} 1}  $$

Similarly, we define *S*_00_(*B*,*D*) as the total number of times corresponding components of *A* and *B* are both 0, i.e., $S_{00}(B,D) = {\sum }_{f=1}^{F} \delta _{{a_{f}^{D}} 0} \delta _{b_{f} 0}$. We also define the “1 − 0 mismatches” in the self-evident way following above: $S_{01}(B,D) = {\sum }_{f=1}^{F} \delta _{{a_{f}^{D}} 0} \delta _{b_{f} 1}$ and $S_{10}(B,D) = {\sum }_{f=1}^{F} \delta _{{a_{f}^{D}} 1} \delta _{b_{f} 0}$.

Let us now define the quantity *S*(*B*,*D*) as the total number of corresponding prefactor-matches minus the total number of prefactor-mismatches between the *D*^*th*^ training vector and test vector *B*, i.e., *S*(*B*,*D*) = *S*_11_(*B*,*D*) + *S*_00_(*B*,*D*) − *S*_10_(*B*,*D*) − *S*_01_(*B*,*D*). We note that SIP was defined as the natural extension of *S*, as the total number of prefactor matches in corresponding regions between the test vector and *all* the training vectors in the class minus the total number of mismatches. Define $\sigma _{11}(B,C) = {\sum }_{D=1}^{W} S_{11}(B,D)$, etc. Thus, the SIP between test *B* and class *C* is written as:

24 $$ \begin{array}{@{}rcl@{}} \sigma(B,C) &= \sigma_{11}(B,C) + \sigma_{00}(B,C) - \sigma_{10}(B,C) - \sigma_{01}(B,C) \end{array} $$

25 $$ \begin{array}{@{}rcl@{}} &= \sum\limits_{D=1}^{W} S(B,D) \end{array} $$

where *W* is the total number of training vectors in the class. We see that *σ* is a natural linear extension of *S*. This linear behavior is retained in the computational basis under certain conditions relevant to our classifier. More specifically, when calculated in the computational basis, the sum of the SIPs of multiple sample vectors is equivalent to the SIP of the sum of the vectors as far as successful classification is concerned. Let us see how this condition of SIP *linearity* is achieved and utilized in the computational basis, with state normalization, for the same above-given sample vectors.

We will use different notation for some of the state vectors in this section compared with previous sections for ease of proof. In moving from feature basis to computational basis now, we will use corresponding Greek notation for state vectors where necessary. We recall that in the SIP framework, for the state vector of any training or test sample, the coefficient for the *f*^*th*^ computational basis vector can only be 1 or − 1 (pertaining to the case where there is a CNV present in the region and the case where there is not, respectively). So, we start with defining the training vector (we will use |*d*〉 to denote the training vector and |*w*〉 to denote the class vector in this section to make the distinction):

26 $$   |{d}\rangle = \frac{1}{\eta_{d}} \left (\sum\limits_{f=1 | {\alpha_{f}^{d}} = 1}^{F} {\alpha_{f}^{d}} |{f}\rangle + \sum\limits_{f=1 | {\alpha_{f}^{d}} = -1}^{F} {\alpha_{f}^{d}} |{f}\rangle \right )  $$

where |*f*〉’s are computational basis vectors as before, ${\alpha _{f}^{d}}$ is the unnormalized *f*^*th*^ component of |*d*〉 in the computational basis, *η*_*d*_ is the overall normalization constant, and *F* again denotes the total number of feature/computational dimensions. The two summations in Eq. 26 serve to pick out only terms where ${\alpha _{f}^{d}}=1$ and ${\alpha _{f}^{d}}=-1$, respectively. Now, the class vector is:

27 $$   |{w}\rangle = \frac{1}{\eta_{w}} \left (\sum\limits_{f=1 | {\alpha_{f}^{d}} = 1}^{F} \sum\limits_{d=1}^{W} {\alpha_{f}^{d}} |{f}\rangle + \sum\limits_{f=1 | {\alpha_{f}^{d}} = -1}^{F} \sum\limits_{d=1}^{W} {\alpha_{f}^{d}} |{f}\rangle \right ) ,    $$

where *η*_*w*_ is the overall normalization constant and *W* is again the total number of training vectors in the class. And finally, the test vector can be written as:

28 $$   |{t}\rangle = \frac{1}{\eta_{t}} \left (\sum\limits_{f=1 | \beta_{f} = 1}^{F} \beta_{f} |{f}\rangle + \sum\limits_{f=1 | \beta_{f} = -1}^{F} \beta_{f} |{f}\rangle \right ),    $$

in exact functional and notational analogy with |*d*〉 in Eq. 26.

Given that the class vector is just a normalized sum over its training vectors, and that in any SIP in the computational basis each matching computational vector component will add + 1 to the result and each mismatching component will add − 1, 〈*t*|*d*〉 is equal to the total number of regional CNV-matches minus the total number of mismatches between the test vector |*t*〉 and training vector |*d*〉, multiplied by their respective normalization constants.

29 $$ \begin{array}{@{}rcl@{}}   \langle{t}|{d}\rangle &=& \frac{1}{\eta_{d} \eta_{t}} \left (S_{11}(B,D) + S_{00}(B,D) - S_{01}(B,D) - S_{10}(B,D) \right ) \end{array} $$

30 $$ \begin{array}{@{}rcl@{}}   &=& \frac{1}{\eta_{d} \eta_{t}} S(B,D) \end{array} $$

We will now calculate 〈*t*|*w*〉, which is the natural computational basis representation of the SIP between *B* and *C*. Since the class vector |*w*〉 is simply a linear sum of all its training vectors and the dot product (state overlap) is a linear operation:

31 $$ \begin{array}{@{}rcl@{}}   {\langle{t}|{w}\rangle} &= \frac{1}{\eta_{w} \eta_{t}} \sum\limits_{D=1}^{W} S(B,D) \end{array} $$

32 $$ \begin{array}{@{}rcl@{}}   &= \frac{1}{\eta_{w} \eta_{t}} \sigma(B,C) \end{array} $$

Note that the difference in normalization constant between the single training vector and the class vector is retained. As *W* can be arbitrary, Eq. 31 proves the implementation of SIP linearity in the computational basis. 5 We see that 〈*t*|*w*〉 is of the form *κσ*(*B*,*C*), for some positive *constant* $\kappa = \frac {1}{\eta _{w} \eta _{t}}$, and hence is a monotonic function of *σ*(*B*,*C*). The fact that *κ* is a constant is seen trivially for the case where there is only one training vector in each class, as $\eta _{w} = \frac {1}{\sqrt {F}}$ for both classes in the SIP framework. For the case of multiple training vectors per class, the form of the data determines the constancy of *κ*. The statistical nature of our neuronal AD/neurotypic contextual data is such that the total number of CNVs in each sample is similar across both disease and normal classes. In fact, the data reveals that the *total* number of CNVs per genomic region has the same overall statistical distribution in each class (van den Bos et al. 2016). This gives rise to the normalization constant (which is a function of these regional coefficients) being the same for both classes in the computational basis. Classification in separable data is still possible due to a salient region where all training samples of a class show atypical activity or different regional arrangements giving rise to the same overall histogram of CNVs per region. As one general example, different floors of large multistory buildings may have different occupancy for different buildings, but the overall *distribution* of floor occupancy could be very similar. At any rate, only the normalization constant needs to be similar for both classes. Any data satisfying this constraint is amenable to SIP classification. This implies that *η*_*w*_, and thus *κ*, are very similar for both classes if the same (or similar) statistically significant number of training vectors is chosen for both, which is natural for our context. When 〈*t*|*w*〉 is used as a classification metric, the higher of two SIPs will remain the higher one irrespective of the value of *κ*. This means that our classifier is valid independent of the value of the normalization constants. Thus, we have shown that our quantum machine successfully executes SIP-based classification and preserves linearity.

There are ways to address the normalization constant issue and are expected to be implemented in future work. As an example, one can encode a simplistic “scaling factor” (ratio of the raw normalization constant of one class vector to the other’s; please see “Efficient data-encoding techniques” for details) using the coefficients of the ground and excited state of a “scaling” qubit. These coefficients would play the role of the raw normalization constants by which the respective class vectors would be multiplied/scaled at run time. Of course, one can multiply this factor classically after the inner product computation, but that would not be a satisfying solution. Without this, if normalization constants for the classes are not similar, correct classification is still possible in a weaker sense. The normalization constant, being the square root of the sum of squares of basis-vector coefficients, is a slowly increasing function of any one coefficient. From the form of the inner product, one can see that if one genomic region in one training class had a particular prevalence of CNVs, classification of a similar test sample into that class would not suffer. In the contextual data, for example, a test sample typically has only 1 CNV in the genome and is highly inclined toward being classified into the class displaying particularly more CNVs in the very same region where that CNV lies.

Finally, we will now prove the equivalence of SIP to the Hamming distance (actually its negative, as mentioned) as a classification metric. In the language of SIP, Eq. 22 can be rewritten by definition as *H*(*B*,*D*) = *S*_01_(*B*,*D*) + *S*_10_(*B*,*D*). We will simply define the Hamming distance *χ* between a test vector and a class vector to be the sum of the Hamming distances between the test vector and each training vector in the class, i.e., $\chi (B,C) = {\sum }_{D=1}^{W} H(B,D)$. As an aside here, we can immediately see how the Hamming distance is not a linear function of the sample vectors as *χ* is not a linear extension of *H*. As a trivial example, the two Hamming distances between 1-d training vectors (1),(0) and test vector (1) add to 1 whereas the Hamming distance between the test vector and the vector sum of the training vectors is 0. Coming back to SIP-Hamming distance equivalence, − *χ*(*B*,*C*) can be rewritten as:

33 $$ \begin{array}{@{}rcl@{}}   - \chi(B,C) &=& - \sum\limits_{D=1}^{W} S_{01}(B,D) + S_{10}(B,D) \end{array} $$

34 $$ \begin{array}{@{}rcl@{}}   &=& - \sum\limits_{D=1}^{W} {F} - S_{11}(B,D) - S_{00}(B,D) \end{array} $$

35 $$ \begin{array}{@{}rcl@{}}   &=& \left (\sum\limits_{D=1}^{W} S_{11}(B,D) + S_{00}(B,D) \right ) - W {F} \end{array} $$

Now, Eq. 25 can be rewritten as:

36 $$ \begin{array}{@{}rcl@{}}   \sigma(B,C) &\!=&\! \sum\limits_{D=1}^{W} S_{11}(B,D) + S_{00}(B,D) - (F - S_{00} (B,D) - S_{11}(B,D)) \end{array} $$

37 $$ \begin{array}{@{}rcl@{}}   &=& 2 \left (\sum\limits_{D=1}^{W} S_{11}(B,D) + S_{00}(B,D) \right ) - W {F} \end{array} $$

We see that Eq. 35 and Eq. 37 are very similar. In each equation, if we fix the number of training vectors per class, the term *WF* can be removed. Now, we see that ${\sum }_{D=1}^{W} S_{11}(B,D) + S_{00}(B,D)$ and $2 {\sum }_{D=1}^{W} S_{11}(B,D) + S_{00}(B,D)$ only differ by a factor of 2 and hence are classificationally equivalent. Thus, we have proven that SIP and Hamming distance are classificationally equivalent measures.

There is one caveat to SIP to mention here. As measurement statistics are always represented by the square of the coefficients/probability amplitudes, 〈*t*|*w*〉 in Eq. 32 will be measured indirectly via |〈*t*|*w*〉|^2^. Thus, the missing information will be whether *σ*(*B*,*C*) in Eq. 24 is positive or negative, i.e., whether the total number of prefactor matches is higher or lower than the number of mismatches. This is addressed via Eqn (2) of (Wiebe et al. 2014a) which shows how to indirectly (semi-classically) arrive at the exact inner product via calculation of cosine similarity between vectors. However, this ambiguity is no issue for the contextual data since each sample has less than 2 CNVs across all genomic regions (van den Bos et al. 2016), ensuring that any test sample will have many more matches (mostly “0 − 0 matches) than mismatches with each training class. For data where individual samples have decidedly fewer 1’s than 0’s, or vice versa, the sign of *σ* will be a priori known. For clarity, we will summarize the cases where SIP-based classification in our circuits cannot succeed:

1. If the raw normalization constants of the two class vectors in feature space are not similar
2. If the data differs by a significant overall scaling factor (see above or see “Efficient data-encoding techniques”)
3. If it is not a priori known whether the number of prefactor matches will be greater or less than the number of prefactor mismatches. However, circuits can be revised by Eqn (2) of (Wiebe et al. 2014a) to address the issue partially.

#### AIP

AIP was defined as the total number of “1 − 1” matches between the test vector and all training vectors composing the class vector for a given class. Note that *σ*_11_(*B*,*C*) in Eq. 24, is exactly the AIP, *A*(*B*,*C*), for test *B* and class *C*. As for SIP, we will again begin with showing how AIP is a bona fide classification metric and prove its linearity. In the AIP framework, we recall that in the state vector of any training or test sample the coefficient for the *f*^*th*^ computational basis vector can only be 1 or 0 (pertaining to the case where there is a CNV present in the region and the case where there is not, respectively). Following the same line of proof as for SIP and retaining the above feature basis definitions (and overall notation), we will now redefine state vectors in the computational basis for the AIP framework. The training vector in the AIP framework reads:

38 $$ \begin{array}{@{}rcl@{}}   |{d}\rangle &= \frac{1}{\eta_{d}} \left (\sum\limits_{f=1 | {\alpha_{f}^{d}} = 1}^{F} {\alpha_{f}^{d}} |{f}\rangle + \sum\limits_{f=1 | {\alpha_{f}^{d}} = 0}^{F} {\alpha_{f}^{d}} |{f}\rangle \right ) \end{array} $$

39 $$ \begin{array}{@{}rcl@{}}   &= \frac{1}{\eta_{d}} \sum\limits_{f=1 | {\alpha_{f}^{d}} = 1}^{F} {\alpha_{f}^{d}} |{f}\rangle \end{array} $$

The test vector can similarly be written as $|{t}\rangle = \frac {1}{\eta _{t}} {\sum }_{f=1 | \beta _{f} = 1}^{F} \beta _{f} |{f}\rangle $. Now, in analogy with SIP:

40 $$   \langle{t}|{d}\rangle = \frac{1}{\eta_{d} \eta_{t}} S_{11}(B,D) $$

and

41 $$ \begin{array}{@{}rcl@{}}   \langle{t}|{w}\rangle &= \frac{1}{\eta_{w} \eta_{t}} \sum\limits_{D=1}^{W} S_{11}(B,D) \end{array} $$

42 $$ \begin{array}{@{}rcl@{}}   &= \frac{1}{\eta_{w} \eta_{t}} \sigma_{11}(B,C) \end{array} $$

As *W* can be arbitrary, Eq. 41 proves the implementation of AIP linearity in the computational basis. Following exactly the same argument as for SIP at this point for the same kind of data, we conclude that the above inner product leads to successful classification. Thus, we have shown that our quantum AIP machine classifies successfully and preserves linearity. We will now show the classificational equivalence of AIP to the negative Hamming distance with the caveat that its equivalence to Hamming distance is not crucial, even undesirable, for classification problems where 1 − 1 matches are more significant than other kinds of prefactor matches. In such cases, like the schedule-matching problem shown in the previous section, AIP is the natural measure of choice. Proceeding with the demonstration of equivalence now, we know that the AIP between test sample *B* and class vector of *C*, $\sigma _{11}(B,C) = {\sum }_{D=1}^{W} S_{11}(B,D)$. We would like to show as above that this is classificationally equivalent to ${\sum }_{D=1}^{W} S_{11}(B,D) + S_{00}(B,D) = \sigma _{11}(B,C) + \sigma _{00}(B,C)$. Clearly, this equivalence is valid when *σ*_00_, the total number of “0 − 0” matches between the test and training vectors of the class in question, is either fixed for both classes somehow, or is a monotonically increasing function of *σ*_11_ in general. The way these two conditions hold true would certainly vary from case to case, so we will simply sketch a few plausible scenarios here. The latter condition is true, for example, when the difference in the number of CNV and non-CNV regions in any given sample is similar to that in other samples (assuming again that the same number of training vectors is chosen for each class). It is also true in the case where CNVs are generally sparsely distributed for all samples. This is most certainly the situation with the contextual data, where any sample statistically has only a single CNV across the whole genome. For test and training samples with sparsely distributed CNVs, an increase in *σ*_11_ is accompanied by an equal increase in *σ*_00_ with a high likelihood. To see how this is the case, one can visualize a test sample vector with a CNV not “in line” (see Fig. 1) with regionally coinciding CNVs of a class’s training vectors, and as soon as it is placed “in line” by swapping it with a non-CNV in another region in the sample, both the “1 − 1” and “0 − 0” matches will very likely simultaneously increase, as long as the likelihood of finding a CNV in the samples is low. Thus, we have shown that our AIP quantum machine is a valid classifier and that it is equivalent to Hamming-distance as a measure under certain conditions, and summarized specific cases satisfying those conditions. We will again enumerate the cases where AIP classification in our circuits will not succeed:

1. If the raw normalization constants of the two class vectors in feature space are not similar
2. If the data differs by a significant overall scaling factor (see Efficient Data-encoding Techniques)
3. If the total number of “0 − 0” prefactor matches is neither fixed for the different classes nor is a monotonically increasing function of the “1 − 1” matches

### Data-encoding techniques

One universal challenge for encoding feature-space data using the computational basis is that one can fix only the relative values of the vector components, as the state vector must be normalized in the computational basis. For example, the unnormalized class vectors *A* = (1,1),*B* = (2,2),*C* = (3,3) in two-dimensional feature space will all be encoded as $S=\frac {1}{\sqrt {2}}(1,1)$ in the computational basis and will not be distinguishable. *A* denotes that there is 1 CNV in each genomic region, *B* denotes 2 in each and *C* denotes 3 in each. If we had two classes with class vectors given respectively by *A* and *B*, the scaling factor (of *B* relative to *A*) would be 2. This is because the *raw normalization constant* for *B* is $2\sqrt {2}$ and that of *A* is $\sqrt {2}$. Thus, we see that the raw normalization constant is nothing but the square root of the sum of the squared number of CNVs in each genomic region: $\sigma = {\sum }_{f=1}^{n} {V^{2}_{f}}$ where *V*_*f*_ is the number of CNVs in region *f* of *n* total genomic regions. One natural way to represent class vectors is $|{d}\rangle = \frac {1}{\sigma }(V_{1},\dots ,V_{n})$. In this *raw vector notation*, class vectors can be juxtaposed for comparing corresponding feature values. If the raw normalization constant is similar for the 2 class vectors, we know that the 2 quantum inner products would be equally scaled relative to the true (unnormalized) inner products in feature space and the classifier would work correctly. Physically, in this simple case, the scaling factor of 2 signifies class *A* having 1 CNV in each of two regions and class *B* having twice as many CNVs in each region. They will both be encoded the same and will not be distinguishable from each other. As mentioned above, there are ways to address this issue in future work such as encoding a scaling factor into the circuit. Fortunately, the kind of data that we are dealing with does not present this issue, again due to the fact that the raw normalization constant for the two classes is very similar if a similar number of training vectors is used. This normalization ambiguity is a current major limitation of our metrics when used for general classification problems without the scaling factor in place. Thus, the metrics are most applicable to data sets whose *overall* scaling across the feature dimensions is similar enough so that the inner product values for the different classes would not be relatively affected. Some data-encoding techniques applicable to our context are presented here. These are not universally applicable for all forms of input data but may be useful to encode data that satisfies certain criteria. The brute force approach is not cheap but is provided to extract broader functionality and for completeness.

#### Brute force approach

It is not in general trivial to encode arbitrary data values from feature space into *n*-qubit systems starting from the universal ground state via a series of available unitary gate operations. A non-trivial combination of ancilla-like entangling qubits and controlled rotations are typically required even for the case of two qubits (see “Data Encoding via Entanglement Routine” in Fig. 5). Broadly, there are two data encoding strategies that can be considered: (1) attempt to directly encode the state coefficients into the quantum computer using a complex set of gate operations which have to be determined or (2) use a classical-quantum approach whereby systems of equations are solved classically that allow simple gate operations to be performed. Many have focused on the challenge of the first approach (Biamonte et al. 2017; Aaronson 2015; Ciliberto et al. 2018; Cortese 2018). Here, we will demonstrate that the second approach is extremely challenging and destroys any advantage associated with the speedup of the quantum algorithm. In our context, an *n*-qubit sample vector is encoded by positive, real state coefficients, a subset of which can be represented in the following (binomial-series-like) state:

43 $$   |{\psi}\rangle = (a_{n}|{0}\rangle + b_{n} |{1}\rangle)...(a_{1}|{0}\rangle + b_{1} |{1}\rangle)  $$

where the *a*_*i*_’s (*i* = 1,2,...*n*) are data-dependent coefficients. Even though this representation is without qubit entanglement, we shall present it to encode sample vectors otherwise represented by entangled states in the computational basis, to be accepted in the case that the final error is low. As a reminder, the combined coefficient of the term |0〉^⊗*n*^ represents the prefactor (CNV value) of the first physical (genomic) region, that of the term |0〉^⊗(*n*− 1)^|1〉 represents the CNV value of the second region, etc. The recipe is as follows:

1. First, the sample vector is normalized in the feature basis using its overall normalization factor $\frac {1}{N}$. Then, expanding terms in Eq. 43 leads to 2^*n*^ non-linear equations in the coefficients *a*_*i*_ and *b*_*i*_ of the form $c_{n} c_{n-1}...c_{1} = \frac {A}{N} \textrm { etc.}$ where the *c*_*i*_’s (*i* = 1,2,...*n*) are placeholders for either *a*_*i*_ or *b*_*i*_, and *A* refers to the relevant regional prefactor/CNV-value. (The case of sample vectors whose natural representation in the computational basis is via entangled states is not readily addressed by this scheme but can be accepted if the error generated in the solution to follow is low.)
2. Next, the non-linear equations are numerically solved for the *a*_*i*_ and *b*_*i*_ in the closest possible form satisfying the constraint that each *i*^*th*^ qubit state term in |*ψ*〉 in Eq. 43 is individually normalized, which automatically leads to the solution |*ψ*〉 being normalized overall. Error minimization techniques can be used to solve these equations for the *a*_*i*_’s and *b*_*i*_’s with the state normalization constraint, possibly containing some error relative to the actual data, especially for naturally entangled states.
3. Finally, each *i*^*th*^ qubit state in Eq. 43 is rotated by *R*_*y*_(*𝜃*) to achieve these minimum error values of *a*_*i*_ and *b*_*i*_.

One example of an entangled state, |*ψ*〉 = *a*_2_*a*_1_|00〉 + *b*_2_*a*_1_|10〉 + *b*_2_*b*_1_|11〉, being represented in the above framework is if the coefficients *a*_2_, *b*_1_ are relatively low and *a*_1_,*b*_2_ are relatively high. This would mean that a term containing *a*_2_*b*_1_ is negligible and |*ψ*〉≈ (*a*_2_|0〉 + *b*_2_|1〉)(*a*_1_|0〉 + *b*_1_|1〉) would be a viable solution. Depending on the specific meaning, purpose, and intended use of the data, one may decide that certain terms or features weigh less in certain contexts to make the above approach feasible. Thus, modulo the scaling factor, this recipe can be used to encode a somewhat broad feature-data space up to some known possible error.

Importantly, solving *N* = 2^*n*^ linear equations is *O*(*N*^3^) in complexity. Solving *N* non-linear equations according to the approach specified in step 2 above is likely much more computationally costly. The approach is provided here to demonstrate the data input challenge in our context and for completeness until other solutions to solve the data input problem, including qRAM, may be realized. These solutions are critical at any rate for the full potential of all quantum computing solutions to be realized. There will necessarily be many cases where a small error will not be achievable (specifically cases where the natural representation of the sample vector in the computational basis is with entanglement may be fraught with high approximation error), rendering this recipe ineffective for such scenarios.

#### Binomial series approach

This is an optimization that can be utilized in both the AIP and SIP frameworks if the data has certain simplifying CNV patterns amenable to this approach. In fact, it was employed in both example problems 1 and 2 of the 14-qubit circuit. However, we again note that this is not a universal solution to simplifying the data input problem. Rather, it is a very useful heuristic approach to simplify the encoding of input data that belongs to a specific “symmetry group,” using minimal gates. We first note that, in the AIP framework, the non-zero coefficients in Eq. 43 will decide which regional blocks are encoded as having CNVs and which ones as not. Therefore, the values of these coefficients can be chosen to encode specific themes or patterns in the data. We will first motivate this in the context of the AIP.

Suppose, for example, that the data has all zero CNV entries in the second half (block) of all genomic regions (i.e., in 32 of 64 regions, etc.). We immediately see that setting *a*_*n*_ = 0 will achieve this condition. If the data has all zero entries in the second half of the first *and* second half blocks of genomic regions, setting *b*_*n*− 1_ = 0 will achieve this. Setting *b*_*n*_,*b*_*n*− 1_ = 0 will enable only states beginning with the term |00...〉 to be present, nullifying all CNV values except those of the first half of all genomic regions and so on. Similarly if it is desired that the CNV values of the first half of all regions be exactly twice that of the second half, one would set *a*_*n*_ = 2 and all the other coefficients to 1 (prior to normalization) and so forth. Many such desired patterns can be creatively effected in data encoding from this general framework. For example, this approach was extended to the SIP framework of example problem 2 of the 14-qubit circuit by finding simple rotations to encode the zero coefficients as “− 1.” This “binomial” series approach combines the idea of using ordered bit strings/ block states to enumerate physical regions with the use of unentangled 1-qubit states juxtaposed to yield a series representation for all unentangled *n*-qubit states in the computational basis. This series representation is then minimalistically utilized for optimal encoding.

### Optimization techniques

#### Swap like-valued bits only in inner product evaluation

When calculating an inner product of two state vectors, each composed of multiple qubits, one need not assess the contribution of corresponding qubits when their contribution is unity. This happens typically when corresponding qubits are like-valued. For example, if |*A*〉 = (*a*_1_|0〉 + *a*_2_|1〉)|0〉|1〉 and |*B*〉 = (*b*_1_|0〉 + *b*_2_|1〉)|0〉|1〉,

$$ \begin{array}{@{}rcl@{}}   \langle{A}|{B}\rangle &= a_{1} b_{1} \langle{0}|{0}\rangle + a_{1} b_{2} \langle{0}|{1}\rangle + a_{2} b_{1} \langle{1}|{0}\rangle + a_{2} b_{2} \langle{1}|{1}\rangle \\   &= a_{1} b_{1} + a_{2} b_{2} \end{array} $$

Clearly, the second and third qubit values of *A* and *B* do not enter the actual evaluation of the inner product, as they are correspondingly identical and contribute a factor of 1 to the result. Of course, if *a*_1_ = *b*_1_ and *a*_2_ = *b*_2_, then the inner product is simply unity by default. One possible way to realize the above operation would be to assess qubit equality by applying a CNOT gate between two corresponding qubits, and then a NOT gate on the target (second) qubit, whose final value serves as the boolean result of the comparison. This value would be designed to trigger the swap test operation for these two qubits by acting as its control. A prior copy of the second qubit would be made in order to preserve the state. For computational basis states, this can be done via a CNOT gate having this second qubit as control and a qubit containing |0〉 as the target, which would be the copy destination. In this way, the state overlap between corresponding qubits would be calculated only when they are different. 6

There will no doubt be better and more efficient ways to determine qubit-state equality and to embed these optimizations within the quantum circuitry in the long term. These assembly-level optimizations could well be realized as a combination of classical and purely quantum methods. We have provided a sketch here. We are currently unable to implement sophisticated circuits anyway due to architectural constraints, etc., and in our work have given a proof of concept for future implementations of the inner product classifier that would employ much more developed quantum hardware and circuitry. One could just as well insert swap gates for like-valued qubits (for computing their trivial overlaps) in order to present “complete” circuits, but we did not think it vital to the theme of this work.

Online Resource 1 presents further optimization techniques not specific to our context.

#### Zero-coefficient exclusion

This is a data-reformulation technique for AIP that saves dimensions in feature space and hence qubits in computational space. Suppose that some of the physical regions in the test vector have a prefactor of 0, thus ensuring that these dimensions will have no contribution to the inner product. The feature vectors can now be mapped onto a new basis so as to eliminate the non-contributing dimensions from the computational framework altogether. For example, say the original test vector residing in 4-dimensional feature space reads $\textbf {x}=\frac {1}{2}(|{00}\rangle _{t} + |{10}\rangle _{t})$, requiring 2 qubits to encode. Clearly, we only need to use feature dimensions 1 and 3 to calculate the AIP. Thus, we eliminate dimensions 2 and 4 from consideration and reformulate our basis such that the dimensional labels 1,3− > 1^′^,2^′^, where the primed dimensions refer to the new basis. The test vector in the new basis now reads $\textbf {x}'=\frac {1}{2}(|{00}\rangle _{t} + |{01}\rangle _{t})$ and only requires 1 qubit to encode. The class vectors are also mapped to the new basis and the AIP is calculated in the usual manner.

Of course, the remapping will change the overall normalization constant for the class vectors. Thus, this technique is limited in scope. The classification outcome is assured to be the same in situations where the overall scaling constant of the two (or more) class vectors changes by the same factor after remapping, leaving the inner product unchanged. This is specifically achieved when the overall scaling factor for the two classes is originally the same and similar number of CNVs are present in the excluded features, or in cases where the classes contain single training samples whose coefficients for most of the features are non-zero (trivially true in the SIP framework). Moreover, correct classification is assured in the case where the overall scaling factor for both classes is the same in the *remapped* basis (please see “Efficient data-encoding techniques” for details and examples of the scaling factor). In the example above, the relative number of CNVs in regions 1 and 3 in the original basis will remain the same for the normal and disease class vectors even after the remapping. If the new scaling factor for both classes is approximately the same, the inner product may change but correct classification would be expected. This can be seen by writing these class vectors in raw notation in the new basis to see that the new inner product would be a true representation of the unnormalized inner product, leading to correct classification (please refer to “Efficient data-encoding techniques” for details).

## Electronic supplementary material

Below is the link to the electronic supplementary material.

(PDF 146 KB )

(PDF 52.9 KB )

(PDF 82.1 KB )

(PDF 316 KB )

(PDF 75.3 KB )

## Footnotes

1 Problems that are solved by the simulator are also solvable by the real processors, which present additional architectural constraints, and we present such solution counterparts for most of the cases.

2 Physically, *𝜃* is the angle of clockwise rotation the normal class vector would undergo from initially pointing along the feature II axis (see Fig. 2) to be thus rendered in the computational basis.

3 $\frac {\pi }{600}$ to be exact

4 The data motivated the development of the classifier but example problems showing specific circuit functionality may certainly be set up with different-looking genomes.

5 The attentive reader will protest here as *η*_*w*_ will change with addition of training vectors. This will not matter for classification if we keep the number of training vectors similar for both classes as seen next.

6 A “copy” of a qubit in superposition using the same method can also be made in the sense that a measurement of the copy would yield the original state measurement probabilities.
