# Supplementary material for: Implementation of a Hamming distance–like genomic quantum classifier using inner products on ibmqx2 and ibmq_16_melbourne
Source: Quantum Mach Intell. 2020 Jul 17;2(1):7. doi: 10.1007/s42484-020-00017-7 (PMC7446251; doi:10.1007/s42484-020-00017-7)
Supplement: Supplementary file 1 — (PDF 146 KB ) [file 42484_2020_17_MOESM1_ESM.pdf]

Implementation of the Swap Test

The Swap Test Explained

The swap test is a quantum algorithm that requires three qubits to assess the extent to which two single qubit quantum states $|\phi\rangle$ and $|\psi\rangle$ differ. Without loss of generality, we can write $|\phi\rangle$ and $|\psi\rangle$ using our notation for disease and test class states

$$|\phi\rangle = \sum_{t_j \in \{0,1\}} c_{t_j} |t_j\rangle_t \tag{1}$$

$$= c_0 |0\rangle_t + c_1 |1\rangle_t , \tag{2}$$

and

$$|\psi\rangle = \sum_{d_j \in \{0,1\}} c^i_{d_j} |d_j\rangle_d \tag{3}$$

$$= c^i_0 |0\rangle_d + c^i_1 |1\rangle_d , \tag{4}$$

where we will demonstrate the relevant swap operation between the matched test qubit state $|t_j\rangle_t$ and train state $|d_j\rangle_d$. The relevant full starting state for the swap operation and test is

$$|0, \phi, \psi\rangle = |0\rangle_s \sum_{t_j \in \{0,1\}} \sum_{d_j \in \{0,1\}} c_{t_j} c^i_{d_j} |t_j\rangle_t |d_j\rangle_d . \tag{5}$$

Application of the Hadamard gate to the swapper qubit $|0\rangle_s$ yields

$$H_s |0, \phi, \psi\rangle = \sum_{t_j \in \{0,1\}} \sum_{d_j \in \{0,1\}} c_{t_j} c^i_{d_j} \frac{1}{\sqrt{2}} \big(|0\rangle_s |t_j\rangle_t |d_j\rangle_d + |1\rangle_s |t_j\rangle_t |d_j\rangle_d \big) . \tag{6}$$

Operation of the CSWAP or Fredkin gate leaves $|\phi\rangle$ and $|\psi\rangle$ (and correspondingly $|t_j\rangle_t$ and $|d_j\rangle_d$) unchanged when the swapper qubit is in the ground state, $|0\rangle_s$, while swapping the $|\phi\rangle$ and $|\psi\rangle$ (and correspondingly $|t_j\rangle_t$ and $|d_j\rangle_d$) states when the swapper qubit is in the excited state, $|1\rangle_s$, giving

$$CSWAPH_s |0, \phi, \psi\rangle = \sum_{t_j \in \{0,1\}} \sum_{d_j \in \{0,1\}} c_{t_j} c^i_{d_j} \frac{1}{\sqrt{2}} \big(|0\rangle_s |t_j\rangle_t |d_j\rangle_d + \tag{7}$$

$$|1\rangle_s |d_j\rangle_d |t_j\rangle_t \big). \tag{8}$$

Applying another Hadamard gates to the swapper qubit results in the final state before measurement

$$H_s CSWAPH_s |0,\phi,\psi\rangle = \sum_{t_j \in \{0,1\}} \sum_{d_j \in \{0,1\}} c_{t_j} c^i_{d_j} \frac{1}{2} \big(|0\rangle_s |t_j\rangle_t |d_j\rangle_d + \tag{9}$$

$$|1\rangle_s |t_j\rangle_t |d_j\rangle_d + \tag{10}$$

$$|0\rangle_s |d_j\rangle_d |t_j\rangle_t - \tag{11}$$

$$|1\rangle_s |d_j\rangle_d |t_j\rangle_t \big). \tag{12}$$

Collecting terms in $|0\rangle_s$ and $|1\rangle_s$ and use of Eqs. (1),(3) results in the well-known pre-measured state of the swap test

$$H_s CSWAPH_s |0,\phi,\psi\rangle = \frac{1}{2} |0\rangle_s \big(|\phi,\psi\rangle + |\psi,\phi\rangle \big) + \frac{1}{2} |1\rangle_s \big(|\phi,\psi\rangle - |\psi,\phi\rangle \big). \tag{13}$$

The probability of measuring the the swapper qubit in state $|0\rangle_s$ is

$$P(|s=0\rangle_s) = \frac{1}{2} + \frac{1}{2} |\langle\psi|\phi\rangle|^2, \tag{14}$$

which is the well-known result of the swap test assessing the extent of overlap of states $|\psi\rangle$ and $|\phi\rangle$ while the probability of measuring the swapper qubit in state $|1\rangle_s$ is

$$P(|s=1\rangle_s) = \frac{1}{2} - \frac{1}{2} |\langle\psi|\phi\rangle|^2 \tag{15}$$

which is the measurement that we use to quantify the inner product between test and train states and ultimately make the classification of the training state into either the normal or disease class.

Understanding the Fredkin Gate Decomposition

As detailed in the manuscript, we implement the controlled-swap or Fredkin gate using 18 elementary IBM Q gates (please refer to Online Resource 4 for the circuit elements). The Fredkin gate consists 1 qubit serving as control and 2 other qubits that are the target of the swap operation. The controlled swap operation has the following functionality: *if the control qubit is in state $|1\rangle$, swap the states held by the* 2 *target qubits; else, do nothing.*

It was shown by (Smolin and DiVincenzo, 1996) that the Fredkin gate can be implemented by placing 2 CNOT gates around the well-known Toffoli (CC-NOT) gate, as shown in Online Resource 4. We use the decomposition of the Toffoli gate that was presented in the Supplement of (Schuld et al., 2017) using 10 single-qubit gates and 6 CNOT gates. In the figure, q_0 is the control qubit and q_1 and q_2 are the target qubits of the swap operation. For the Toffoli gate, q_0 and q_1 act as controls and q_3 acts as the target of the CCNOT operation.

Of course, the Fredkin gate treats qubits q_2 and q_3 identically in terms of functionality. We show here for a generic state how the Fredkin gate operation is executed by the gate sequence $CNOT \cdot CCNOT \cdot CNOT$. In terms of the qubits shown in Online Resource 4, we execute the controlled swap operation on the generic 2-qubit state $\left|\psi\right\rangle_{q_1 q_2} = (a_1\left|0\right\rangle + b_1\left|1\right\rangle)_{q_1}(a_2\left|0\right\rangle + b_2\left|1\right\rangle)_{q_2}$ twice, first with q_0 being in state $\left|0\right\rangle_{q_0}$ and then in state $\left|1\right\rangle_{q_0}$. We show that the first execution does not alter $\left|\psi\right\rangle_{q_1 q_2}$ whereas the second one swaps the individual states held by q_1 and q_2. Denoting by C_X^{ij} the CNOT gate with qubit q_i as control and qubit q_j as target, and by CC_X^{ijk} the CCNOT gate with qubits q_i and q_j in control and qubit q_k acting as target, the CSWAP operation reads:

$$CSWAP\left|\psi\right\rangle_{q_1 q_2} = C_X^{12} CC_X^{321} C_X^{12}\left|\psi\right\rangle_{q_1 q_2} \tag{16}$$

In case 1 where q_0 is in state $\left|0\right\rangle_{q_0}$, CC_X^{321} effectively reduces to the identity operator I. Thus, Eq. (16) becomes

$$\begin{aligned} CSWAP\left|\psi\right\rangle_{q_1 q_2} &= C_X^{12} C_X^{12}\left|\psi\right\rangle_{q_1 q_2} \\ &= \left|\psi\right\rangle_{q_1 q_2} \end{aligned}$$

and nothing is swapped as claimed. In case 2, q_0 is in the state $\left|1\right\rangle_{q_0}$ and CC_X^{321} reduces to C_X^{21} yielding

$$\begin{aligned} CSWAP\left|\psi\right\rangle_{q_1 q_2} &= C_X^{12} C_X^{21} C_X^{12}\left|\psi\right\rangle_{q_1 q_2} \\ &= C_X^{12} C_X^{21} C_X^{12}(a_1\left|0\right\rangle + b_1\left|1\right\rangle)_{q_1}(a_2\left|0\right\rangle + b_2\left|1\right\rangle)_{q_2} \\ &= C_X^{12} C_X^{21} C_X^{12}(a_1 a_2\left|0\right\rangle_{q_1}\left|0\right\rangle_{q_2} + a_1 b_2\left|0\right\rangle_{q_1}\left|1\right\rangle_{q_2} \\ &\quad + b_1 a_2\left|1\right\rangle_{q_1}\left|0\right\rangle_{q_2} + b_1 b_2\left|1\right\rangle_{q_1}\left|1\right\rangle_{q_2}) \\ &= C_X^{12} C_X^{21}(a_1 a_2\left|0\right\rangle_{q_1}\left|0\right\rangle_{q_2} + a_1 b_2\left|0\right\rangle_{q_1}\left|1\right\rangle_{q_2} \\ &\quad + b_1 a_2\left|1\right\rangle_{q_1}\left|1\right\rangle_{q_2} + b_1 b_2\left|1\right\rangle_{q_1}\left|0\right\rangle_{q_2}) \\ &= C_X^{12}(a_1 a_2\left|0\right\rangle_{q_1}\left|0\right\rangle_{q_2} + a_1 b_2\left|1\right\rangle_{q_1}\left|1\right\rangle_{q_2} \\ &\quad + b_1 a_2\left|0\right\rangle_{q_1}\left|1\right\rangle_{q_2} + b_1 b_2\left|1\right\rangle_{q_1}\left|0\right\rangle_{q_2}) \\ &= (a_1 a_2\left|0\right\rangle_{q_1}\left|0\right\rangle_{q_2} + a_1 b_2\left|1\right\rangle_{q_1}\left|0\right\rangle_{q_2} \\ &\quad + b_1 a_2\left|0\right\rangle_{q_1}\left|1\right\rangle_{q_2} + b_1 b_2\left|1\right\rangle_{q_1}\left|1\right\rangle_{q_2}) \\ &= a_2\left|0\right\rangle_{q_1}(a_1\left|0\right\rangle_{q_2} + b_1\left|1\right\rangle_{q_2}) + b_2\left|1\right\rangle_{q_1}(a_1\left|0\right\rangle_{q_2} + b_1\left|1\right\rangle_{q_2}) \\ &= (a_2\left|0\right\rangle_{q_1} + b_2\left|1\right\rangle_{q_1})(a_1\left|0\right\rangle_{q_2} + b_1\left|1\right\rangle_{q_2}) \\ &= (a_2\left|0\right\rangle + b_2\left|1\right\rangle)_{q_1}(a_1\left|0\right\rangle + b_1\left|1\right\rangle)_{q_2} \end{aligned}$$

Thus, in case 2, the generic states held by qubits q_1 and q_2 are swapped by the Fredkin gate. Q.E.D.

Useful Techniques and Observations

This section contains generally useful observations or techniques that the authors stumbled upon, but were not directly applied to our inner product circuits.

Quantum Compression Technique

One can *encode* an arbitrary large number m using merely 2 qubits as follows. Prepare a state $|\psi\rangle = a|0\rangle + b|1\rangle$ such that $a/b = k$, and another state $|\phi\rangle = c|0\rangle + d|1\rangle$ such that $c/d = l$, where $l \leq 1$ and $m = l2^k$. Thus m is a fraction, given by l, of the largest value that a binary string of k bits can hold in the usual base-2 representation. Of course, k could in principle be directly used to store the desired large value, but k and l are both used here for better precision. The success of quantum compression necessarily depends on the precision achievable in state preparation (i.e. in rotation angles). Classically, there is no way around storing the precise number itself in some form whereas here we can effectively compress it into one precisely prepared qubit. The measurement would constitute the decoding/decompression.

Calculating Hamming Distance with XOR-based Schemes

We used the feature basis to encode our example data, but will point out here that the most seemingly natural way to calculate the Hamming distance in a quantum computer is to use bit-wise CNOT (or XOR) gates applied between bit strings that are encoded directly as a series of $|0\rangle$ and $|1\rangle$ states in the computational basis. For example, the Hamming distance between "001" and "111" is given by the bit string "110," which is of course output of $|001\rangle \oplus |111\rangle$. Measuring the coefficients of all 3-qubit basis states after this CNOT operation would reveal $|110\rangle$ as the final result. However, this way to calculate the Hamming distance is highly inefficient in general, as each bit occupies a qubit and each training vector has to be separately encoded.

Inner-product Decision Plane for Multiple Classes

For multiple training classes, the decision boundary for the inner product classifiers is not a simple plane or hyperplane as it is for two classes. In order to construct the decision space, one would draw a bisector plane for each class, dividing the feature space into preferred subregions by class, and pick the class that is the universally preferred class in the region where the test vector lies. For example, on a plane, one could have 3 class vectors for classes 1, 2 and 3 respectively, yielding a total of 3 bisecting planes or lines. These lines divide the feature space into "$R-1 > R-2$", "$R-2 > R-3$" etc. subregions for

a total of six such overlapping subregions (see Fig. 2). Here, $R-1 > R-2$ means that class 1 is preferred over class 2 for that subregion. Each point in the feature space will be part of exactly two overlapping subregions where a particular class (1,2 or 3) is preferred over the other two. So the point where test vector lies determines its classification without any ambiguity in this manner. Please refer to Online Resource 5 for the figure.

References

Schuld, M., Fingerhuth, M., and Petruccione, F. (2017). Implementing a distance-based classifier with a quantum interference circuit. *EPL (Europhysics Letters)*, 119(6):60002.

Smolin, J. A. and DiVincenzo, D. P. (1996). Five two-bit quantum gates are sufficient to implement the quantum fredkin gate. *Phys. Rev. A*, 53:2855–2856.
