# Supplementary figures and images for: Implementation of a Hamming distance–like genomic quantum classifier using inner products on ibmqx2 and ibmq_16_melbourne
Source: Quantum Mach Intell. 2020 Jul 17;2(1):7. doi: 10.1007/s42484-020-00017-7 (PMC7446251; doi:10.1007/s42484-020-00017-7)

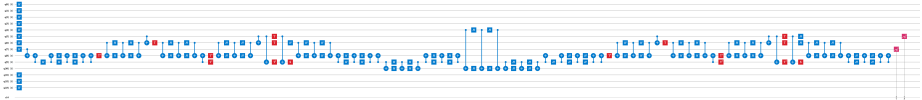

**Supplementary Figure 2:** 14-qubit Example Problem 1 Circuit on IBMQX16

Supplement: Supplementary file 3 — (PDF 82.1 KB ) [file 42484_2020_17_MOESM3_ESM.pdf]
