# Supplementary material for: Implementation of a Hamming distance–like genomic quantum classifier using inner products on ibmqx2 and ibmq_16_melbourne
Source: Quantum Mach Intell. 2020 Jul 17;2(1):7. doi: 10.1007/s42484-020-00017-7 (PMC7446251; doi:10.1007/s42484-020-00017-7)
Supplement: Supplementary file 4 — (PDF 316 KB ) [file 42484_2020_17_MOESM4_ESM.pdf]

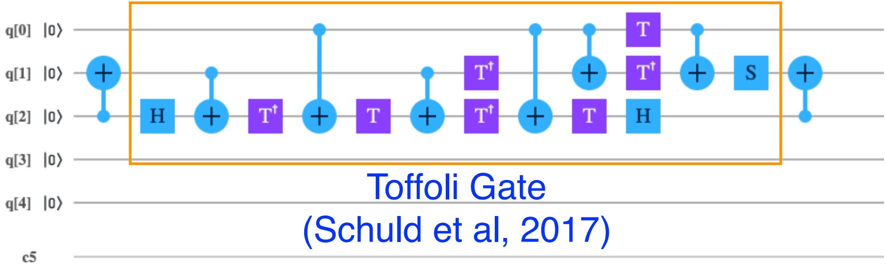

Supplementary Figure 3: Components of the Fredkin gate implementation we employed are shown in terms of IBM Q native operations, sourced from IBM Quantum Experience. Qubit q_0 is in control and swaps q_1 and q_2.
