# Supplementary material for: Implementation of a Hamming distance–like genomic quantum classifier using inner products on ibmqx2 and ibmq_16_melbourne
Source: Quantum Mach Intell. 2020 Jul 17;2(1):7. doi: 10.1007/s42484-020-00017-7 (PMC7446251; doi:10.1007/s42484-020-00017-7)
Supplement: Supplementary file 5 — (PDF 75.3 KB ) [file 42484_2020_17_MOESM5_ESM.pdf]

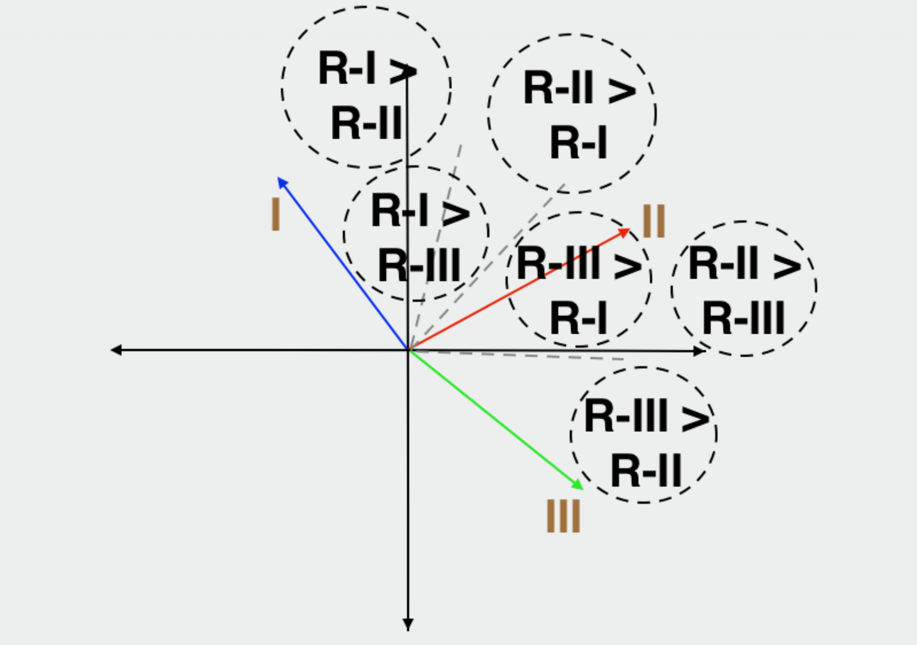

Supplementary Figure 4: Preferred decision subregions for > 2 classes. The class vectors are shown in red, blue and green and indicated in roman numerals, whereas the grey dotted lines are the respective bisectors of each pair of class vectors. The dotted circles label the preferred subregions.
