# Supplementary material for: Implementation of a Hamming distance–like genomic quantum classifier using inner products on ibmqx2 and ibmq_16_melbourne
Source: Quantum Mach Intell. 2020 Jul 17;2(1):7. doi: 10.1007/s42484-020-00017-7 (PMC7446251; doi:10.1007/s42484-020-00017-7)
Supplement: Supplementary file 2 — (PDF 52.9 KB ) [file 42484_2020_17_MOESM2_ESM.pdf]

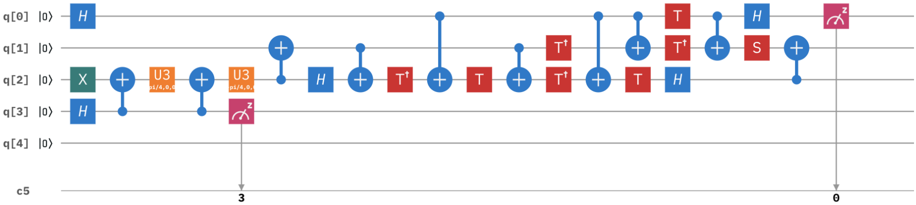

Supplementary Figure 1: 5-qubit Example Problem 1 Simulation Circuit
